# User Interface Designs to Address Cancer Misinformation: A Randomized Clinical Trial

**DOI:** 10.1001/jamanetworkopen.2026.41122

**Published:** 2026-10-29

**Authors:** Allison J. Lazard, Mirian Avendaño-Galdamez, Tara Licciardello Queen, Shelby Lake, Tushar Varma, David Marshall, Hung-Jui Tan, Jacob N. Stein, Nabarun Dasgupta

**Affiliations:** 1 Hussman School of Journalism and Media, University of North Carolina at Chapel Hill, Chapel Hill; 2 Lineberger Comprehensive Cancer Center, University of North Carolina at Chapel Hill, Chapel Hill; 3 Department of Health Behavior, Gillings School of Global Public Health, University of North Carolina at Chapel Hill, Chapel Hill; 4 Injury Prevention Research Center, University of North Carolina at Chapel Hill, Chapel Hill; 5 Department of Urology, School of Medicine, University of North Carolina at Chapel Hill, Chapel Hill; 6 Division of Oncology, School of Medicine, University of North Carolina at Chapel Hill, Chapel Hill

## Abstract

**Importance:**

Misinformation about false and potentially harmful cancer treatments is common on social media. Strategies are needed to encourage reporting to reduce exposure to inaccurate treatment claims.

**Objective:**

To evaluate the impact of 2 user interface designs (prompts on posts) on intervening and sharing cancer misinformation on social media.

**Design, Setting, and Participants:**

This 3-group, parallel-group, randomized clinical trial assessed outcomes at 5 time points from March 31 to May 12, 2025. US adults (ages 18-65 years) who had a cancer diagnosis or identified as a cancer caregiver and used social media were recruited from Prolific. Participants interacted with cancer misinformation posts on a simulated realistic social media platform. Data were analyzed from May 13, 2025, to July 27, 2026.

**Interventions:**

Participants were randomized to cancer misinformation posts shown with social cue–enhanced prompts that specify others’ actions and a review policy, standard prompts, or no prompts (control).

**Main Outcomes and Measures:**

The primary outcome was a participant flagging a post to intervene. Primary and secondary outcomes included recorded social media behavior (flagging, disliking, liking) and self-reported willingness to flag, dislike, like, comment, and share about the posts. Intervening outside the study platform was also assessed. A separate generalized estimating equations model was used for each outcome.

**Results:**

A total of 294 individuals (mean [SD] age, 39.3 [11.7] years; 179 [62%] women) with cancer or who were caregivers for individuals with cancer were randomized, with 88 participants receiving social cue prompts, 92 receiving standard prompts, and 114 receiving no prompts. Posts with inaccurate claims were flagged 2 to 3 times more often with social cue (224 of 592 posts [38%]) and standard (180 of 689 posts [26%]) prompts than those without prompts (84 of 675 posts [12%]) (social cue vs control: rate ratio [RR], 3.12 [95% CI, 1.79-5.42]; standard vs control: RR, 2.15 [95% CI, 1.20-3.88]). Prompts led to less sharing behavior (ie, liking: 20 posts [3%] with social cue prompts; 10 posts [1%] with standard prompts) compared with posts without prompts (65 posts [10%]) (social cue vs control: RR, 0.07 [95% CI, 0.04-0.15]; standard vs control: RR, 0.13 [95% CI, 0.04-0.42]). Prompts also led to greater willingness to flag and dislike (intervening) and less willingness to like and share compared with no prompt. Standard prompts led to more flagging on existing social media (outside the study platform) than no prompts.

**Conclusions and Relevance:**

In this randomized clinical trial of user interface designs, simple prompts encouraged responsible behavior online. Prompts on posts led to more intervening and less sharing of cancer misinformation.

**Trial Registration:**

ClinicalTrials.gov Identifier: NCT07588607

## Key Points

**Question:**

Can simple changes to user interface designs encourage intervening to reduce inaccurate cancer treatment claims on social media?

**Findings:**

In this randomized clinical trial with 294 individuals with cancer or caregivers of individuals with cancer, social cue and standard social media prompts led to double or triple the number of posts flagged to report to the social media platform.

**Meaning:**

These findings suggest that user interface designs with simple prompts on posts encouraged meaningful action to report and reduce exposure to false and potentially harmful cancer misinformation.

## Teaser

This randomized clinical trial evaluates whether social cue and standard prompts changes rates of flagging, disliking, liking, and sharing social media posts with cancer misinformation.

## Introduction

Inaccurate claims about cancer treatments are widely shared on social media,^1,2,3^ and most individuals with cancer have exposure to this misinformation.^4,5^ Cancer claims that are false, misleading, or unsupported by current scientific evidence are misinformation.^6,7,8^ Inaccurate claims about cancer treatments are psychologically harmful by causing distress, false hope, and disrupting critical social support (eg, abandoning resources if confronted with misinformation).^9,10^ Inaccurate cancer claims can also be physically harmful. Patients can experience harm if their caregivers are distressed, and patients who follow unsupported treatments or refuse clinical recommendations may have greater disease progression or mortality.^11,12,13,14^ In our online communication landscape, where 1 in 5 individuals with cancer report making health-related decisions based on social media,^4^ addressing cancer misinformation is necessary to prevent harm.

Although some inaccurate claims are shared maliciously (eg, profit from the uncertainty and fear that often come with a cancer diagnosis), misinformation is also shared by individuals with cancer and loved ones.^15,16^ Redirecting sharing to intervene on inaccurate cancer claims can reduce exposure and harms. Intervening through 1-click challenges (eg, flagging) on social media is a small, albeit powerful action if done by many.^17^ User-flagged posts can be hidden, deemphasized in feeds, or brought to the attention of moderators.

Prompts (also called *labels*, *warnings*, or *nudges*) on social media posts can encourage intervening and reduce sharing misinformation about cancer^15,18^ and other topics.^19,20,21,22,23,24,25,26^ Prompts with standard, topic-agnostic language (eg, “This post has been flagged as false and potentially harmful. If you want to report, click the flag below.”) alert viewers and provide guidance for action—necessary steps for intervening according to human-computer interaction adaptions of the bystander intervention model.^27,28,29,30,31^

Prompts may be more effective with social cues and clear polices for platform responses. Supported by social presence theory,^32^ social cues about others’ actions provide a subtle but effective way to increase attention and prosocial behavior online (eg, showing that 47 people have flagged encourages flagging behavior).^18,33^ However, to our knowledge, no trials have examined whether social cue prompts or standard prompts (vs no prompts) lead to more intervening with cancer misinformation over time.

To examine the impact of prompts across time, we conducted a 5–time point, randomized, parallel-assignment trial using a simulated social media platform. We hypothesized that social cue prompts and standard prompts would lead to more intervening with inaccurate cancer claims than no prompts and that social cue prompts would lead to more intervening than standard prompts. We asked whether the impact of prompts changed over time and whether prompts (social cue and standard vs control) reduced sharing. To contextualize actions, we assessed reactions to cancer support posts without misinformation. Last, we explored whether prompts influenced intervening behavior outside of the study platform.

## Methods

### Procedure

We conducted a 3-group, parallel-group, randomized clinical trial with 1:1:1 allocation in a superiority framework from March 31 to May 12, 2025. Intervention exposure and outcome assessment occurred across 5 time points among adults with cancer or caregivers of individuals with cancer. The study was exempt by the University of North Carolina institutional review board (No. 25-0269), preregistered at AsPredicted,^34^ and registered on ClinicalTrials.gov. All participants provided consent online. The trial protocol and statistical analysis plan are available in Supplement 1. This study is reported following the Consolidated Standards of Reporting Trials (CONSORT) reporting guideline.

Participants were recruited via Prolific, an online, opt-in panel of verified individuals for academic research,^35,36,37^ with a short message about our upcoming 5 surveys for an academic research study about cancer posts shared on social media and link to the screener survey. The target sample size was 300 individuals (100 per group) based on our primary outcome power analysis (trial protocol and statistical analysis plan in Supplement 1). US adults were eligible if they were aged between 18 and 65 years, had received a cancer diagnosis and/or been a caregiver for an individual with cancer at any age, used social media, and were willing to complete all 5 study assessments in English. After data collection began, we added a preview of our simulated social media to the screener survey to ensure individuals did not have technical issues prior to enrollment. Participants received up to $35.50 for completing the screener and 5 time point surveys.

Eligible individuals provided consent by reading an online consent form and selecting to agree to participate before beginning the time 1 (T1) survey. Participants completed surveys on Qualtrics. Participant sociodemographic characteristics, including race and ethnicity, were collected by self-report. Race and ethnicity were categorized as American Indian or Alaska Native, Asian, Black or African American, Hispanic or Latino, Middle Eastern or North African, multiracial, White, or another race or ethnicity (not otherwise specified). Race and ethnicity were assessed to allow for recruitment that reflected demographics in the US.

At T1, participants were randomized to a trial group with cancer misinformation posts with social cue prompts and policy (social cue group), with standard prompts (standard group), or without prompts (control). Simple randomization (1:1:1) in Qualtrics, using a pseudorandom number generator in embedded data, allocated participants across the 3 groups. Participants knew their allocated trial group from stimuli exposure but were blind to the study objectives or hypotheses. At each time point, participants were exposed to 2 cancer misinformation posts and 1 cancer support post in 2 ways (passive and forced exposure). First, participants interacted with stimuli on our simulated social media platform, Invibe, embedded in the Qualtrics survey (passive exposure). Second, on the following survey webpages, participants viewed the stimuli as static posts in a random order and answered self-reported items (forced exposure). Participants were debriefed at the end of each survey with the following: “Please note that we developed the social media posts in this survey for research purposes. The social media posts you saw were not evidence-based cancer recommendations.” Surveys were fielded every 2 business days and closed before the following time point. Study personnel contacted participants through Prolific with survey reminders and technical or incentive support. There was no involvement from patients or the public outside of the study participants.

### Stimuli

#### Cancer Misinformation Posts

Each time point’s Invibe feed of 80 posts included 2 cancer misinformation posts (total of 10 posts across the trial), 1 cancer support post (5 posts across the trial), and 77 background posts (385 posts across the trial) (stimuli available in the trial protocol in Supplement 1). Cancer misinformation posts were adapted from content commonly shared on image-focused social media (eg, miracle cures, distrust of the medical industry).^5,38,39,40,41^ Posts were vetted by oncologists to ensure claims were not supported by current scientific evidence or promising as treatments (eg, no viable mechanism). Cancer misinformation posts were the second and fifth posts at T1, T3, and T5 and the third and sixth posts at T2 and T4.

#### Social Media Prompts

Participants randomized to view the experimental groups (social cue or standard) were shown prompts on 2 cancer misinformation posts at each time point (Figure 1). Prompts were on the post image above the caption, with a warning icon (exclamation in triangle) to increase attention.^26,42^ Prompts were not shown on the cancer misinformation posts for the control group. Prompts were not shown on cancer support posts or any background posts.

**Figure 1.**
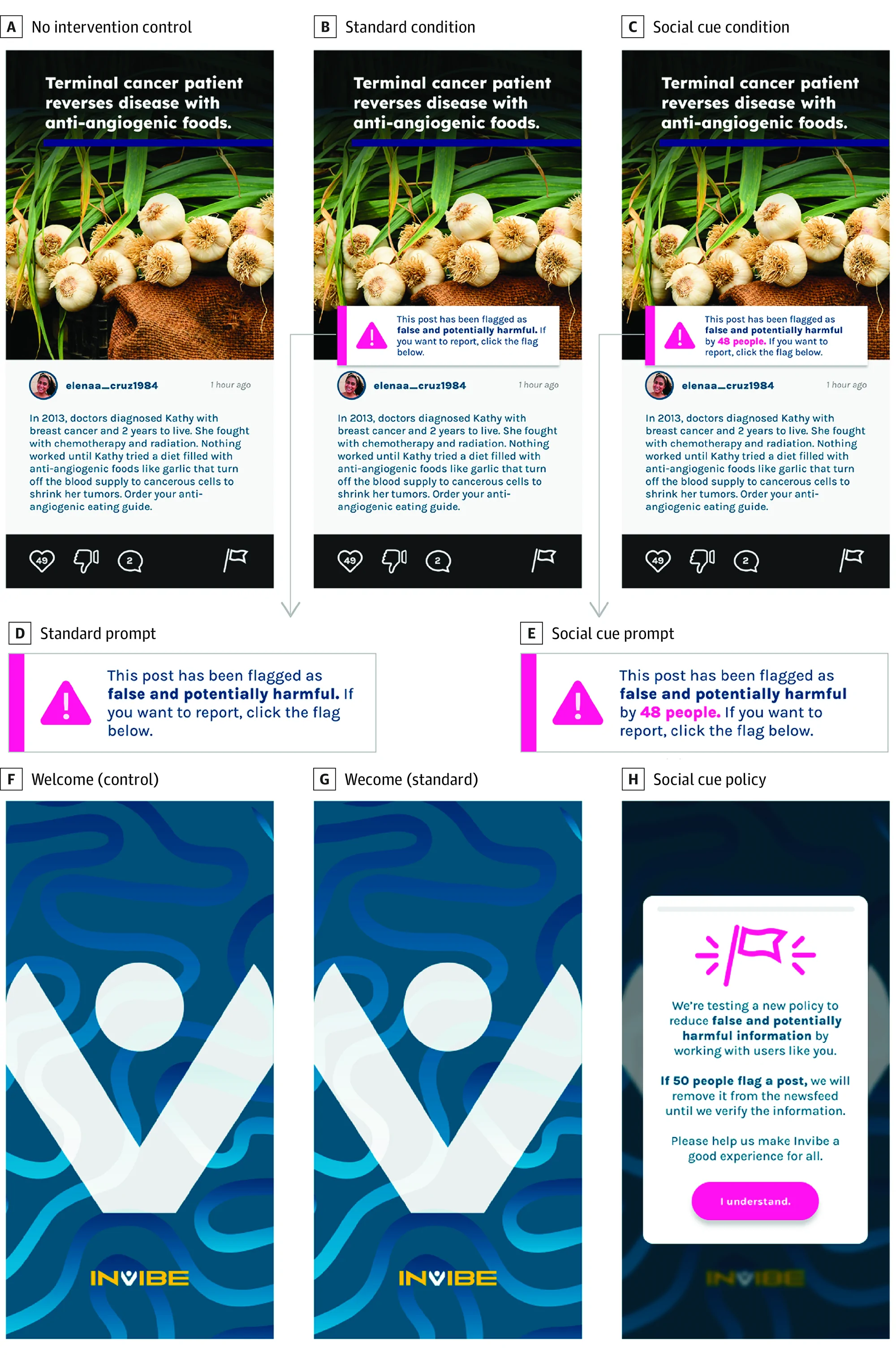
Prompts and Policy

For the standard group, the prompt stated: “This post has been flagged as false and potentially harmful. If you want to report, click the flag below” (Figure 1B and D), similar to other studies.^19,20,21,22,23,24,25,26^ For the social cue group, others’ actions were added: “This post has been flagged as false and potentially harmful by [47-48] people. If you want to report, click the flag below” (Figure 1C and E). For the social cue group, the launch screen (logo only) was overlaid with a policy for removing flagged posts: “We’re testing a new policy to reduce false and potentially harmful information by working with users like you. If 50 people flag a post, we will remove it from the newsfeed until we verify the information. Please help us make *Invibe* a good experience for all.” (Figure 1H). With 47 to 48 previous flags, participants’ flagging was near the tipping point but not the deciding action for post review.

#### Cancer Support Posts

To descriptively compare social media behavior (passive exposure) and self-reported outcomes (forced exposure), we assessed posts designed to mimic helpful cancer support (trial protocol in Supplement 1).^9^ Such posts were positioned as the fourth post on each Invibe feed.

### Measures

#### Intervening and Sharing Behavior

We captured intervening with flagging as participants selecting the flag icon (primary outcome) and disliking as participants selecting the thumbs down icon. We captured sharing with liking as participants selecting the heart icon. Behaviors were separately dichotomized for each stimuli post (coded as 1) or no action (0).

#### Willingness to Intervene and Share

Participants reported willingness to flag (“How willing are you to flag this post?”), dislike (“How willing are you to dislike (thumbs down) this post?”), like (“How willing are you to like (heart) this post?”), comment (“How willing are you to comment on this post?”), and share (“How likely are you to share or tell someone about this post?”). Responses ranged from not at all (coded as 1) to extremely (5). Participants reported reactions to 2 cancer misinformation posts and 1 cancer support post at each time point.

Since comments could intervene or share, we asked comment valence (“What type of comment would you write on this post?”), with response options of comment to agree (share), comment to disagree (intervene), other, or would not comment. At T1 and T5, we also asked participants with whom they would share (“Who would you share about this post with?”) and how (“How would you share about this post?”).

#### Past-Week Intervening

To assess intervening outside of our simulated platform, at T4, we asked “In the last 7 days, did you take action against false and potentially harmful cancer posts from social media?” We captured the intervening behavior, social media platform or intervening recipient, and topic (eTable 1 in Supplement 2).

### Statistical Analysis

To examine the effects of trial group and time on each social media behavior (binary) and willingness outcomes (continuous), we conducted a separate generalized estimating equations (GEE) model for each outcome (trial protocol and statistical analysis plan in Supplement 1). Each GEE model had trial group as a categorical predictor (social cue, standard, control), time as a repeated measure across 5 time points, and the interaction between trial group and time. For the mixed-effects models, all available observations were included in the intent-to-treat analyses without imputation. Our preregistration specified that we would dichotomize our binary outcomes per time point. After data collection, we opted to retain each behavioral outcome (2 per time point) because action on a greater number of posts is more meaningful than regular action. We used a consistent approach and entered measures of each continuous outcome in the models. We used a critical α = .05 or 95% CIs that did not cross the null, with 2-tailed tests. The overall observed means for each trial group are reported in the main tables with rate ratios (RRs) or mean differences and 95% CIs; model means and trial group comparisons by time for social media behaviors and willingness outcomes are in eTable 2 and eTable 3 in Supplement 2.

We descriptively report frequencies for participants’ comment valence and with whom and how they would share the posts. To assess trial group differences for intervening outside the simulated platform in the past week, we conducted logistic regressions. We conducted 1 model for each intervening outcome with trial group as the categorical predictor. Data were analyzed using SPSS version 32.0 (IBM) and SAS version 9.4 (SAS Institute) from May 13, 2025, to July 27, 2026.

## Results

A total of 294 participants (mean [SD] age, 39.3 [11.7] years; 179 [62%] women) were enrolled and randomized, including 92 participants (31%) with a previous cancer diagnosis and 257 participants (87%) who served as a caregiver to someone with cancer (Table 1), with 55 participants (19%) reporting both a cancer diagnosis and caregiving experience. By race and ethnicity, there were 1 American Indian or Alaska Native participant (<1%), 14 Asian participants (5%), 78 Black or African American participants (27%), 13 Hispanic or Latino participants (4%), 1 Middle Eastern or North African participant (<1%), 18 multiracial participants (6%), and 167 White participants (57%).

**Table 1.** Participant Characteristics

Characteristic	Participants, No. (%)			
	Overall (N = 294)	Social cue prompt (n = 88)	Standard prompt (n = 92)	No prompt control (n = 114)
Age, mean (SD), y	39.3 (11.7)	39.5 (12.3)	40.3 (11.4)	38.3 (11.3)
Race or ethnicity (n = 293)				
American Indian or Alaska Native	1 (<1)	0	0	1 (1)
Asian	14 (5)	1 (1)	6 (7)	7 (6)
Black or African American	78 (27)	25 (29)	20 (22)	33 (29)
Hispanic or Latino	13 (4)	3 (3)	4 (4)	6 (5)
Middle Eastern or North African	1 (<1)	0	1 (1)	0
Multiracial	18 (6)	5 (6)	7 (8)	6 (5)
White	167 (57)	52 (60)	54 (59)	61 (54)
Another race or ethnicity	1 (<1)	1 (1)	0	0
Gender (n = 291)				
Woman	179 (62)	58 (67)	55 (60)	66 (58)
Man	110 (38)	27 (31)	36 (39)	47 (42)
Nonbinary	2 (1)	1 (1)	1 (1)	0
Transgender				
Yes, transgender	9 (3)	3 (3)	2 (2)	4 (4)
No, not transgender	282 (96)	83 (94)	89 (97)	110 (96)
Prefer not to answer	3 (1)	2 (2)	1 (1)	0
Cancer history (n = 293)^a^				
Yes, in the last 5 y	44 (15)	14 (16)	17 (19)	13 (11)
Yes, >5 y ago	48 (16)	16 (18)	14 (15)	18 (16)
No	201 (69)	58 (66)	60 (66)	83 (73)
Cancer caregiver^b^				
Yes, in the last 5 y	146 (50)	36 (41)	45 (49)	65 (57)
Yes, >5 y ago	111 (38)	40 (45)	34 (37)	37 (32)
Not a cancer caregiver	37 (13)	12 (14)	13 (14)	12 (11)
Social media use^c^				
Several times a day	269 (91)	82 (93)	79 (86)	108 (95)
About once a day	12 (4)	3 (3)	7 (8)	2 (2)
A few times a week	11 (4)	3 (3)	6 (7)	2 (2)
Every few weeks	1 (<1)	0	0	1 (<1)
Less often	1 (<1)	0	0	1 (<1)
Political affiliation				
Republican	123 (42)	38 (43)	37 (40)	48 (42)
Democrat	109 (37)	32 (36)	37 (40)	40 (35)
Independent	56 (19)	17 (19)	16 (17)	23 (20)
Something else	6 (2)	1 (1)	2 (2)	3 (3)

^a^ Participants who reported a previous cancer diagnosis identified the type of cancer they were first diagnosed with, stage at diagnosis, and current treatment status. Reported cancers included bladder (n = 1); brain tumor (n = 2); breast (n = 24); colorectal (n = 3); esophageal (n = 1); gynecologic (n = 11); head and neck (n = 2); kidney (n = 6); leukemia, lymphoma, or other blood cancer (n = 8); liver (n = 4); lung (n = 9); melanoma (n = 11); pancreatic (n = 4); prostate (n = 6); sarcoma (n = 4); testicular (n = 3); thyroid (n = 6); or other (n = 20). Reported stages included I (n = 68), II (n = 35), III (n = 13), IV (n = 4), or not applicable (n = 15). Reported treatment status included completed treatment (n = 85), currently receiving treatment (n = 29), between treatments (n = 11), have not started treatment (n = 4), in hospice care (n = 2), or other (n = 4).

^b^ Participants reported being cancer caregivers for their parents (n = 133), another family member (n = 112), friend (n = 70), spouse or partner (n = 31), child or children (n = 13), or other (n = 10).

^c^ Participants reported using YouTube (n = 232), Instagram (n = 220), Facebook (n = 216), Twitter/X (n = 170), Reddit (n = 168), TikTok (n = 163), Snapchat (n = 105), or other social media (n = 16).

Participants were randomized to the social cue (88 participants), standard (92 participants), or control (114 participants) groups (Figure 2). Participation was high across the 5 time points (T2: 276 participants [94%]; T3: 267 participants [91%]; T4: 259 participants [88%]; T5: 259 participants [88%]), with 278 participants (95%) completing 3 or more time points. Most participants viewed the stimuli via passive exposure (eTable 4 in Supplement 2) before forced exposure. Among 259 participants who rated the study at T5, 256 (99%) agreed it was easy to participate, 259 (100%) would enroll again, and 255 (99%) would recommend the study to a friend.

**Figure 2.**
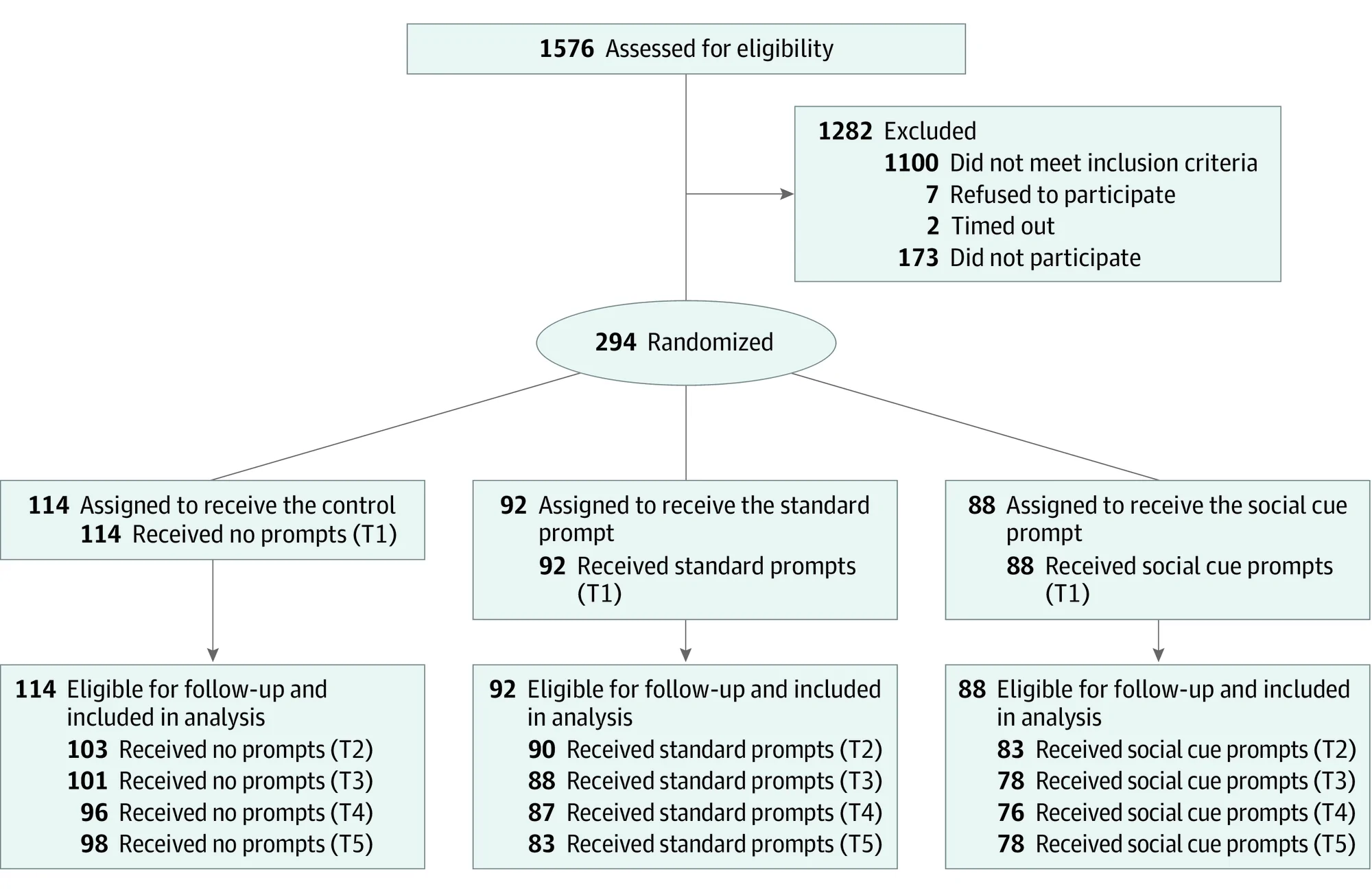
Study Enrollment and Assessment Flowchart T indicates time point.

### Impact on Intervening and Sharing Behavior

Prompts and policies led to significantly more intervening behavior (ie, flagging) among individuals with cancer and caregivers (Table 2). Misinformation posts were flagged 2 to 3 times more often with social cue prompts (224 of 592 posts [38%]) and standard prompts (180 of 689 posts [26%]) than without prompts (84 of 675 posts [12%]) (social cue vs control: RR, 3.12 [95% CI, 1.79-5.42]; standard vs control: RR, 2.15 [95% CI, 1.20-3.88]). Social cue and standard prompts led to similarly elevated flagging (Table 2). The prompts did not affect disliking. Participants intervened by disliking similarly with the social cue prompts, standard prompts, and no prompts. In the same feeds, very few (<1%) cancer support posts were flagged (5 of 1020 posts) or disliked (6 of 1020 posts).

**Table 2.** Impact of Interventions on Social Media Behavior and Willingness to Intervene and Share

Outcome	Prompt			Cancer support posts for comparison (n = 294)	Estimate (95% CI)		
	Social cue (n = 88)	Standard (n = 92)	None (n = 114)		Social cue vs none	Standard vs none	Social cue vs standard
**Social media behavior, No. of posts/total No. of posts (%)**							
Flagging	224/592 (38)	180/689 (26)	84/675 (12)	5/1020 (<1)	3.12 (1.79 to 5.42)^a^	2.15 (1.20 to 3.88)^a^	1.45 (0.97 to 2.17)^a^
Disliking	82/592 (14)	128/689 (19)	70/675 (10)	6/1020 (<1)	1.31 (0.66 to 2.60)^a^	1.82 (0.92 to 3.60)^a^	0.72 (0.42 to 1.24)^a^
Liking	20/592 (3)	10/689 (1)	65/675 (10)	271/1020 (27)	0.07 (0.04 to 0.15)^a^	0.13 (0.04 to 0.42)^a^	0.57 (0.17 to 1.94)^a^
**Self-reported willingness to, mean (SD) score**							
Flag	3.59 (1.63)	3.66 (1.56)	2.48 (1.62)	1.22 (0.53)	1.11 (0.76 to 1.47)^b^	−0.06 (−0.44 to 0.32)^b^	1.18 (0.82 to 1.53)^b^
Dislike	3.48 (1.64)	3.64 (1.58)	2.47 (1.62)	1.28 (0.54)	1.01 (0.65 to 1.37)^b^	−0.17 (−0.55 to 0.22)^b^	1.17 (0.82 to 1.52)^b^
Like	1.57 (1.20)	1.44 (1.05)	2.34 (1.58)	3.96 (0.86)	−0.76 (−1.06 to −0.46)^b^	0.13 (−0.12 to 0.38)^b^	−0.89 (−1.18 to −0.61)^b^
Comment	2.24 (1.46)	2.32 (1.49)	2.53 (1.52)	2.98 (1.15)	−0.29 (−0.63 to 0.05)^b^	−0.08 (−0.43 to 0.27)^b^	−0.21 (−0.54 to 0.12)^b^
Share	1.75 (1.19)	1.57 (1.08)	2.33 (1.53)	2.95 (1.14)	−0.58 (−0.87 to −0.29)^b^	0.18 (−0.07 to 0.42)^b^	−0.76 (−1.04 to −0.48^b^)

^a^ Expressed as rate ratio (95% CI).

^b^ Expressed as mean difference (95% CI).

Social cue and standard prompts led to less sharing behavior (liking) than the control (social cue vs control: RR, 0.07 [95% CI, 0.04-0.15]; standard vs control: RR, 0.13 [95% CI, 0.04-0.42]). While 1 in 10 cancer misinformation posts were liked without prompts, there was a 3-fold decrease in liking with either prompt vs control (Table 2). The effect on liking was moderated by time; less liking was not consistent across time. For context, 1 in 4 cancer support posts were liked across the trial.

### Impact on Willingness to Intervene or Share

Prompts led to greater willingness to flag misinformation compared to no prompts (social cue vs control: mean difference, 1.11 [95% CI, 0.76 to 1.47]; standard vs control: mean difference, 1.18 [95% CI, 0.82 to 1.53]). Social cue and standard prompts did not differ on willingness to flag (Table 2). Participants were somewhat to moderately willing to flag cancer misinformation posts with prompts, compared with being slightly willing without prompts. Time moderated this effect; direction and significance were consistent, but the effect size varied.

Social cue and standard prompts led to greater willingness to dislike and lower willingness to like cancer misinformation posts compared with the control (Table 2). Social cue and standard prompts did not differ on willingness to dislike or like. Again, the direction and significance were consistent at each time point, but the magnitude of effects differed.

There was no impact of prompts on willingness to comment. However, comment valence varied by trial group. Descriptively, participants who received no prompts would comment to agree more often (70 of 114 participants [59%]) than those who received social cue prompts (28 participants [24%]) or standard prompts (20 participants [17%]). Participants would comment to disagree similarly with social cue prompts (65 participants [31%]), standard prompts (72 participants [34%]), and without prompts (76 participants [36%]).

Willingness to share about misinformation posts was lower with social cue and standard prompts than without prompts. Across all 294 participants, 74 (25%) would not share with anyone and 79 (27%) would not share via any channel for any cancer misinformation post at T1 and T5. Others would share at least 1 post with someone with cancer (128 participants [44%]), caregivers (136 participants [46%]), or health care practitioners (113 participants [38%]), with few participants noting they would share with other people (34 participants [12%]). Participants would share at least 1 post by reposting on social media (111 participants [38%]), social media private messages (110 participants [37%]), in person (109 participants [37%]), or text message (99 participants [34%]).

### Impact on Past-Week Intervening

More than half of participants reported intervening with cancer misinformation posts outside the study platform in the past week (Table 3). Disliking posts was most common, followed by flagging posts and muting accounts. There was a main effect of standard prompts for past-week flagging. Participants with standard prompts had 2 times higher odds of flagging cancer misinformation than those without prompts (odds ratio [OR], 2.24 [95% CI, 1.19-4.22]). The social cue group did not differ from the control group (OR, 1.13 [95% CI, 0.57-2.27]). There were no other differences for past-week intervening.

**Table 3.** Participant-Reported Intervening in the Past Week, Assessed at Time 4

Self-reported intervening	Participants, No. (%)			
	Overall (n = 259)	Social cue prompt (n = 76)	Standard prompt (n = 87)	Control, no prompt (n = 96)
Disliked posts	89 (34)	25 (33)	35 (40)	29 (30)
Flagged posts	79 (31)	20 (26)	36 (41)^a^	23 (24)^a^
Muted accounts	58 (22)	15 (20)	22 (25)	21 (22)
Commented to correct posts	41 (16)	11 (15)	12 (14)	18 (19)
Chatted with others to disagree with post information	31 (12)	9 (12)	8 (9)	14 (15)
Encouraged someone to flag or report posts	30 (12)	5 (7)	14 (16)	11 (12)
Did not intervene	119 (46)	38 (50)	36 (41)	45 (47)

^a^ Statistically significantly different at *P* < .05.

When intervening with misinformation outside the study platform, participants intervened on commonly used social media (eTable 5 in Supplement 2). Participants most often intervened about alternative cancer treatments, advice to stop clinical care, or diet and nutrition advice.

## Discussion

In this randomized clinical trial, we found simple changes to user interface designs encouraged flagging to report problematic posts and reduced sharing. Exposure to inaccurate cancer claims on social media could be mitigated by prompts for 1-click actions.^15,18^ As legacy mega-platform content moderation is scaled back, user-driven flagging can be an inexpensive and easily implemented solution to attenuate harm.^2,3^

Prompts on posts led to 2 to 3 times more misinformation posts flagged for review. Across each of the 5 time points over 2 weeks, both social cue and standard prompts effectively encouraged people to intervene on inaccurate cancer claims. Although we anticipated the social presence theory–informed social cues (eg, number who flagged) would amplify high flagging like other studies,^15,18^ it is encouraging that both prompt strategies were highly effective, which suggests flexibility in scalable designs.

The prompts had a desirable spillover effect for reducing sharing, with a 3-fold decrease in liking misinformation posts with prompts. The prompts also reduced willingness to like or share in the future, connecting to literature that prompts and simple rebuttals reduce misperceptions and sharing across many topics.^19,20,21,22,23,24,25,26,43^ Importantly, our prompts for cancer misinformation did not create a problematic chilling effect for sharing cancer support; cancer support posts were often liked on our social media feeds. This is key, given that social media can meet support needs for individuals with cancer.^9,44^

Our experiment builds on evidence that simple, easily implemented designs to deemphasize harmful information can shape the future of social media.^45,46^ While content flagging is available on most platforms and 1-click challenges have been found to reduce the stress and discomfort of online confrontation,^17,47,48^ there is little evidence-based design encouragement for their use. Mega-platforms may test changes based on academic research slowly, but moderated community-run and federated platforms have greater control and can implement these strategies efficiently with adaptions (eg, embedding prompts and policies in posts, comments, or moderation practices)^49^ or through direct deployment (eg, administrative-controlled user interface modifications).^50^

Given the proliferation of inaccurate cancer claims and increasing concerns for the safety of individuals on social media,^51^ user interface designs can function as safeguards for cancer and other misinformation^2^ amid other strategies. In our social media environment with high virality, one celebrity’s endorsement of an off-label cancer treatment spread widely and corresponded with significant increases in prescribing rates of drugs that are not supported by or have yet to be evaluated in rigorous clinical trials.^13^ We need multifaceted efforts to provide individuals with cancer, their caregivers, content moderators, and platform administrators with the tools to increase health literacy (eg, user interface designs and patient-centered messaging that make it easier to find, understand, and apply accurate cancer information)^52^ and encourage trust in health information (eg, narratives from clinicians about the impact of misinformation or plain-language communication from scientists).^53,54^

### Limitations

This study has some limitations. The findings are limited to the types and order of inaccurate cancer claims in the stimuli, our prompts and policy, and participants recruited in our convenience sample. Different stimuli exposure order or content (eg, with some accurate information) may yield different findings. Because of substantial overlap in cancer experiences (ie, survivor and caregiver), we were unable to conduct moderation analyses; future studies should compare findings among individuals with distinct cancer experiences. We used a simulated social media platform to increase internal validity and to protect participants. Future studies should replicate this work on existing platforms. Our repeat testing may have introduced bias; however, the consistent directionality of effects across time points suggests that prompts led to in-the-moment responses rather than learned behaviors from the trial design. We also recognize that prompts do not fully convert people to act by flagging. While prompts led to more flagging, misinformation posts were not flagged more than half the time with prompts, and sharing with individuals with cancer was still common. With evidence for the realistic bounds for user-driven moderation, user interface designs should be paired with other interventions and policies to reduce harm.

## Conclusions

In this randomized clinical trial, we found evidence for 2 readily implemented user interface designs to address cancer misinformation. Prompts (ie, warnings) on posts directed individuals to flag harmful content and discouraged sharing. Prompts that displayed others’ actions tripled flagging to report cancer misinformation compared with no prompt.

## Notes

Supplement 1. Trial Protocol and Statistical Analysis Plan

Supplement 2. eTable 1. Survey items for self-reported willingness to intervene and share eTable 2. Model means and condition comparisons by time for social media behavior eTable 3. Model means and condition comparisons by time for willingness to intervene or share eTable 4. Stimuli exposure on Invibe (passive social media exposure) eTable 5. Platforms and topics for past week intervening

Supplement 3. Data Sharing Statement
