# Supplementary material for: User Interface Designs to Address Cancer Misinformation: A Randomized Clinical Trial
Source: JAMA Netw Open. 2026 Oct 29;9(10):e2641122. doi: 10.1001/jamanetworkopen.2026.41122 (PMC13720694; doi:10.1001/jamanetworkopen.2026.41122)
Supplement: Supplementary file 1 [file jamanetwopen-e2641122-s001.pdf]

STUDY PROTOCOL

Social media posts about cancer:

A randomized controlled trial of user interface designs to encourage intervening and reduce sharing of cancer treatment misinformation

Principal Investigator

Allison J. Lazard, PhD

Co-Investigators

Nabarun Dasgupta, PhD
Jake Stein, MD, MPH
Hung-Jui Tan, MD, MSHPM

Biostatistician

Tara Licciardello Queen, PhD

Research team

Mirian Avendaño-Galdamez, BA
David Marshall, BA
Tushar Varma, BA
Shelby Lake, BA

Study site. University of North Carolina at Chapel Hill

Protocol version. 5.6.2026

LIST OF ABBREVIATIONS

FDA = United States Food and Drug Administration

RCT = Randomized Controlled Trial

N = Sample size

n = Sub-sample size

RESEARCH SYNOPSIS

Study title. Social media posts about cancer

Study population. Adults with a cancer diagnosis or adult cancer caregivers who use social media.

Study design. Parallel randomized controlled trial (RCT).

Primary objective. To examine whether user interface designs (social cue prompts and policy, standard prompts) lead to more intervening of cancer misinformation.

Secondary objectives. To examine whether user interface designs lead to less sharing over time and whether the impact of prompts change over time.

Exploratory objectives. To explore whether user interface designs influence intervening outside of the study social media platform. To descriptively contextualize primary and secondary objective finding with behavior and reactions for cancer support posts.

BACKGROUND AND RATIONALE

Cancer misinformation exerts a heavy burden on cancer patients, survivors, and care networks.[1,2] Cancer misinformation about unproven, potentially harmful treatments is often shared on social media, where misinformation spreads faster and further than factual content.[3,4] Misinformation represents upwards of 30-77% of all cancer information shared on social media with a higher percentage of misinformation in treatment-related content (over 80% in one study of gynecologic cancer).[5-7] Cancer misinformation increases exposure to messages for untested therapies; home or marketing remedies, supplements, or diets; and non-FDA approved providers, facilities, or clinical trials.[2,5,8,9] Exposure to misinformation leads to psychological harm, including distress, decisional regret, or inability to cope.[2] Patients may have worse health outcomes if they act on misinformation, such as stopping chemotherapy, or if caregivers are distressed.[2] Individuals with cancer have an increased risk of death if they abandon clinically recommended care for false cures[10,11] and addressing misinformation to improve treatment decisions could increase survival by more than five times among some cancers.[10-12]

Cancer misinformation about treatment and cures is often spread through cancer care networks of family and friends,[9] likely with good intentions, but is harmful.[13,14] This misguided altruism should be redirected to support community action to intervene. Our highly interactive communication landscape reinforces the immediacy of sharing cancer misinformation, without deliberation. Most U.S. adults own or access a smartphone (85%),[15] are online constantly or multiple times daily (85%),[16] and avidly use visual-based social media (81%).[17] Strategies to redirect community action to intervene for cancer misinformation and prevent sharing reduce negative impacts for those in cancer treatment and survivorship are needed.[18-21]

Social media user interface designs, including in-platform prompts and policies, can reduce cancer misinformation by encouraging pro-social intervening (e.g., flagging, reporting). Evidence-based human-computer interaction extensions of the Bystander Intervention Model[22-26] articulate steps for prosocial intervening on social media including the noticing the potential for harm and seeing a path for action before they can intervene. Social media designs, including prompts and policies, garner attention by alerting for the presence of misinformation and provide clear mechanisms for action. Social media designs to reduce misinformation are beginning to appear on social media. For example, Twitter is expanding a program to encourage flagging of misleading tweets after over 3 million users flagged or reported misinformation in an August 2021 pilot[27] and using Birdwatch to encourage communities to fact check misinformation.[28,29] While there are emerging interventions and strategies to reduce sharing or misperceptions of general health misinformation,[30-32] there are currently no interventions for cancer treatment misinformation. Our goal is to reduce cancer misinformation on social media through prompts with policies addressing false cancer treatments and cures. Our main hypothesis is that social media user interface designs can encourage prosocial intervening with cancer misinformation.

STUDY DESIGN

Study population. Participants were recruited on Prolific, an online survey platform with over 150,000 active panelists.

Inclusion criteria. Participants are eligible if they:

Are currently ages 18-65
Received a cancer diagnosis or have been a cancer caregiver
Use social media
Willing to complete all five study timepoints
Reside in the United States

Exclusion criteria. Individuals are not eligible if they:

Were unable to scroll to see posts on Invibe in the screener survey (updated April 22, 2025, after 31 participants were enrolled)

Our trial is open to all cancer types and treatment stages, an approach that allows those with rare cancers to participate and paves the way to broad dissemination. We set upper limits for female (65%) and non-Hispanic white individuals (65%) to guard against oversampling these groups based on their historically greater participation in research.

Recruitment and screening. We recruited convenience sample of up to 300 adults with cancer or who served as cancer caregivers on Prolific. We aimed to recruit up to 300 participants to have 80% power to detect a small effect (Cohen's f =.20) or larger for differences in flagging between our prompts and no-prompt control, according to our a priori power analysis.

Participants (N = 294) were recruited in two waves to allow for technical issues to be detected and resolved after initial study launch. Recruitment began on March 31, 2025, for the first wave of participants (n = 31). One technical issue was noted and resolved before the launch of second wave (see April 22, 2025 update below). Recruitment began on April 22, 2025, for the second wave of participants (n = 263).

To recruit participants, a screener survey was made available on Prolific to individuals who reported their age between 18-65 years old and that they resided in the United States in their panel profile. Interested individuals received $.50 for completing the one-minute screener survey.

The screener survey appeared on the Prolific site with the following message:

> "This is a short survey (~1 min) to determine if people who are interested are eligible to participate in our upcoming 5 surveys for an academic research study about cancer posts shared on social media. In this short survey you will be asked to share basic information about yourself (e.g., demographics, social media use). Select the link below to complete the survey. You may only take this survey one time."

Updated April 22, 2025: Before launching the second wave of recruitment, we added a preview our simulated social media platform, branded as *Invibe,* to the screener survey to invite individuals without technical issues. Participants were instructed to scroll through a short feed (5

posts). Participants who were unable to scroll to see posts before the initial post were not eligible for participation.

Completed screener surveys were reviewed by the study team to ensure participants met the eligibility criteria (age, had a cancer diagnosis or experience as a cancer caregiver, use social media, willing to complete to all five study surveys, and were able to scroll to see Invibe posts).

Enrollment and retention. Eligible individuals were invited to enroll in the study by starting the first survey. The invitation to begin the study read:

> “This study is about cancer posts shared on social media. **This research includes 5 surveys.**
>
> We’d like to hear from you if are **willing to participate in 5 surveys over the next 3 weeks** and share what you think about the way cancer information is shared online. You can expect a new survey every two business days.
>
> Today’s survey is the first of 5 surveys. You received $5.00 for each 11-minute survey. The 5th survey will include an additional $10.00, so that you will receive $15.00 for the final (5th) ~11-minute survey. You will receive a total of $35.00 for completing all 5 of the 11-minute surveys.
>
> This survey will be available **[time frame]** for you.”

To enroll in the study, individuals clicked the clicked the link to start the timepoint (Time) 1 online survey. All individuals provided consent online before beginning the Time1 survey and subsequent trial. All study surveys were made available to participants via Prolific.

Participants were messaged through the Prolific platform each time a new survey is available, along with reminder messages for uncompleted surveys, as needed. Messages for the next available survey read:

> “Thank you for taking our “Social media posts about cancer” survey. The next available survey is ready for you. It should appear on your dashboard. This will be available from **[dates]**. Here is a direct link to the study for easy reach: https://app.prolific.com/studies/**[study number on Prolific]**
>
> It is a $5.00 payment for this up to 11-minute survey. We appreciate your willingness to share your thoughts.”

Prolific reminder message for participants who have not completed the survey read:

> “Hello! Friendly reminder that “Social media posts about cancer **[Day/Time #]**” survey is available and ready for you to complete. It should appear on your dashboard. This will be available until [date and time]. If you think you will need more time, please let us know. Here is a direct link to the study for easy reach: https://app.prolific.com/studies/[**study number on Prolific]**

It is a **[incentive amount]** payment for this up to 11-minute survey. We appreciate your willingness to share your thoughts."

Timeline.

Wave 1 dates and study steps (*n* = 31)

Day, Date	Study steps
Monday, March 31, 2025	Screener survey recruitment
Tuesday, April 1, 2025	Invite eligible individuals to timepoint (Time) 1 survey
Thursday, April 3, 2025	Close Time 1 survey, invite all enrolled participants to Time 2 survey
Friday, April 4, 2025	Reminder message for Time 2 survey
Monday, April 7, 2025	Close Time 2 survey, invite all enrolled participants to Time 3 survey
Tuesday, April 8, 2025	Reminder message for Time 3 survey
Wednesday, April 9, 2025	Close Time 3 survey, invite all enrolled participants to Time 4 survey
Thursday, April 10, 2025	Reminder message for Time 4 survey
Friday, April 11, 2025	Close Time 4 survey, invite all enrolled participants to Time 5 survey
Monday, April 14, 2025	Reminder message for Time 5 survey
Tuesday, April 21, 2025	Closed study

Wave 2 dates and study steps (*n* = 263)

Day, Date	Study steps
Tuesday, April 22, 2025	Screener survey recruitment
Thursday, April 24, 2025	Invite eligible individuals to timepoint (Time) 1 survey
Friday, April 25, 2025	Close Time 1 survey, invite all enrolled participants to Time 2 survey
Monday, April 28, 2025	Reminder message for Time 2 survey
Tuesday, April 29, 2025	Close Time 2 survey, invite all enrolled participants to Time 3 survey
Wednesday, April 30, 2025	Reminder message for Time 3 survey
Thursday, May 1, 2025	Close Time 3 survey, invite all enrolled participants to Time 4 survey
Friday, May 2, 2025	Reminder message for Time 4 survey
Monday May 4, 2025	Close Time 4 survey, invite all enrolled participants to Time 5 survey
Tuesday, May 5, 2025	Reminder message for Time 5 survey
Monday, May 12, 2025	Closed study

Procedure. Eligible individuals were invited to join the study through a Qualtrics link to the first timepoint survey (Time 1). Time 1 began with an online consent form where participants acknowledged they had read the consent form, were voluntarily participating, were over 18 years old, and had been a cancer caregiver or received a cancer diagnosis.

Consented individuals were randomized with simple randomization to one of three conditions using the Qualtrics randomizer with embedded data: a social media feed with cancer misinformation posts with social cue prompts and policy (social cue prompts and policy), with standard prompts (standard prompts), or without prompts (control). Participants knew their trial arm from stimuli exposure (not blinded) but were not told of randomized trial arms.

Before interacting with our simulated social media platform, *Invibe*, at Time 1, participants were told they were viewing a "beta" platform with instructions for how to scroll through posts on their smartphone, tablet, or desktop computer. *Invibe* has posts and features like other visual-based social media (e.g., Instagram, TikTok), but unbeknownst to the participant, they were the only

real person on the platform. All other people, posts, and reactions were programmed by the research team. Unlike experiments on existing social media, our simulation allowed us to have the control of an experimental lab study (e.g., every participant starts at the same timepoint, no interference with platform algorithms or the day's newsworthy events) combined with the realism of current platforms.

At the beginning of each timepoint survey (Time 1-5), participants interacted with our simulated social media platform, *Invibe*, for **passive exposure** to the study stimuli. On the following pages of the survey, participants viewed the stimuli posts again as static images for **forced exposure**.

> **Passive exposure.** At each timepoint, participants interacted with an *Invibe* feed, with an initial launch screen (welcome logo or review policy) that auto-advanced to a scrollable feed with the stimuli posts (shown via passive exposure in the first six posts) among 80 background posts. The social cue flagging policy was shown for 15 seconds unless participants clicked forward. Participants were reminded to take as much time as they need to explore the feed before moving forward in the study. In wave 2, we added instructions to scroll to view at least 2 posts on *Invibe* and an item on the next survey page to assess how easy or hard it was to scroll to encourage use of *Invibe* and detect any technical issues.
>
> **Forced exposure.** On the following survey webpages, participants viewed static versions of the two cancer misinformation stimuli posts (via forced exposure) and one cancer support post at each timepoint. The static versions were identical to the post shown in the *Invibe* feed. The misinformation and support posts were presented in a random order. Participants viewed one post and answered the self-reported items about this post before viewing the next post at that timepoint.

At the end of the survey participants shared more about their demographics and cancer characteristics (Time 1), social media use (Time 2), past week intervening behavior outside of *Invibe* (Time 4), and perceived usability of the study (Time 1 and Time 5).

All participants were invited to the next timepoint on the same schedule, regardless of a missed survey, after completing Time 1. Participants were invited to complete a survey every two business days. After one business day, a reminder was sent to any participants who had yet to complete that timepoint survey (see timeline above). Each timepoint survey was closed before invitations were sent for the following survey.

Participants received $5 for each 11-minute timepoint survey, along with a $10 bonus for the final (Time 5) survey, for up to $35.50 in incentives for the study.

Intervention. Participants were randomly assigned to one of three trial arms of the RCT at the beginning of the Time 1 survey.

> **Experimental: Social cue prompts and policy.** Cancer misinformation posts are shown with a prompt above the post caption that reads, "This post has been flagged as false and potentially harmful by [47 or 48] people on *Invibe*. If you want to report, click the flag below." The social media launch screen has an overlaid policy for removing flagged posts: "We are committed to working with you and others on Invibe to reduce

false and potentially harmful information. When a post is flagged by 50 people, we remove it until we verify the information. Please help us make the Invibe experience a good one for all."

Experimental: Standard prompts. Cancer misinformation posts are shown with a prompt above the post caption that reads, "This post has been flagged as false and potentially harmful. If you want to report, click the flag below."

Control. No prompts are shown on cancer misinformation posts.

OUTCOME MEASURES

Primary outcomes

1. **Flagging.** Measured with intervening behavior, defined as the flag icon selected. Response options are no (coded as 0) or yes (coded as 1). Assessed up to 15 times. Assessed for 2 cancer misinformation posts and 1 cancer support post at each timepoint approximately 2 business days apart, starting on the day of enrollment.

2. **Willingness to flag.** Measured with 1 self-reported survey item: “How willing are you to flag this post?” Responses ranged from “not at all” (coded as 1) to “extremely” (5). Assessed up to 15 times. Assessed for 2 cancer misinformation posts and 1 cancer support post at each timepoint approximately 2 business days apart, starting on the day of enrollment.

Secondary outcomes

3. **Disliking.** Measured with intervening behavior, defined as the thumbs down icon selected. Response options are no (coded as 0) or yes (coded as 1). Assessed up to 15 times. Assessed for 2 cancer misinformation posts and 1 cancer support post at each timepoint approximately 2 business days apart, starting on the day of enrollment.

4. **Liking.** Measured with intervening behavior, defined as the heart icon selected. Response options are no (coded as 0) or yes (coded as 1). Assessed up to 15 times. Assessed for 2 cancer misinformation posts and 1 cancer support post at each timepoint approximately 2 business days apart, starting on the day of enrollment.

5. **Willingness to dislike.** Measured with 1 self-reported survey item: “How willing are you to dislike (thumbs down) this post?” Responses ranged from “not at all” (coded as 1) to “extremely” (5). Assessed up to 15 times. Assessed for 2 cancer misinformation posts and 1 cancer support post at each timepoint approximately 2 business days apart, starting on the day of enrollment.

6. **Willingness to like.** Measured with 1 self-reported survey item: “How willing are you to like (heart) this post?” Responses ranged from “not at all” (coded as 1) to “extremely” (5). Assessed up to 15 times. Assessed for 2 cancer misinformation posts and 1 cancer support post at each timepoint approximately 2 business days apart, starting on the day of enrollment.

7. **Willingness to comment.** Measured with 1 self-reported survey item: “How willing are you to comment on this post?” Responses ranged from “not at all” (coded as 1) to “extremely” (5). Assessed up to 15 times. Assessed for 2 cancer misinformation posts and 1 cancer support post at each timepoint approximately 2 business days apart, starting on the day of enrollment.

8. **Willingness to share.** Measured with 1 self-reported survey item: “How likely are you to share or tell someone about this post?” Responses ranged from “not at all” (coded as 1) to “extremely” (5). Assessed up to 15 times. Assessed for 2 cancer misinformation posts and 1 cancer support post at each timepoint approximately 2 business days apart, starting on the day of enrollment.

9. **Comment valence.** Measured with 1 self-reported survey item: “What type of comment would you write on this post?” Response options were “comment to agree” (share), “comment to disagree” (intervene), “other,” or “I would not comment.” Assessed up to 15 times. Assessed for 2 cancer misinformation posts and 1 cancer support post at each timepoint approximately 2 business days apart, starting on the day of enrollment.

10. **Sharing recipient.** Measured with 1 self-report survey item: “Who would you share about this post with?” Response options were “someone with cancer,” “someone who is a cancer caregiver,” “health-care provider,” “other,” or “no one.” Assessed up to 6 times. Assessed for 2 cancer misinformation posts and 1 cancer support post at first timepoint and last timepoint approximately 2 weeks apart.

11. **Sharing channel.** Measured with 1 self-report survey item: “How would you share about this post?” Response options were “private message on social media,” “text message,” “in person,” “repost on your social media,” “other,” and “none of these ways.” Assessed up to 6 times. Assessed for 2 cancer misinformation posts and 1 cancer support post at timepoint 1 and timepoint 5 approximately 2 weeks apart.

Exploratory outcomes

12. **Past week intervening behavior.** Measured with 1 self-reported survey item: “In the last 7 days, did you take action against false and potentially harmful cancer posts from social media? Select all that apply.” Response options included muted accounts, flagged posts, disliked posts, commented to correct posts, chatted with others to disagree with posts information, encouraged someone to flag or report a posts, other or did not do anything. Assessed 1 time. Assessed at timepoint 4 approximately a week after the day of enrollment.

13. **Past week intervening platform.** Measured with 1 self-reported survey item per reported intervening behavior on social media: “On which platform(s) did you [intervening behavior]? Select all that apply.” “Response options were provided only when the platform afforded the corresponding intervening action and included platforms where the behavior could occur. Response options could include: Facebook, Instagram, Snapchat, Twitter/X, YouTube, Reddit, or other. Assessed up to 4 times. Assessed at timepoint 4 approximately a week after the day of enrollment.

14. **Past week intervening recipient.** Measured with 1 self-reported survey item per reported interpersonal intervening behavior: “Who did you [intervening behavior]? Select all that apply.” Response options were “someone with cancer,” “someone who is a

cancer caregiver," "health-care provider," "other," or "no one." Assessed up to 2 times. Assessed at timepoint 4 approximately a week after the day of enrollment.

15. **Past week intervening topic.** Measured with 1 self-reported survey item per reported intervening behavior: "What was the [source for intervening] about? Select all that apply." Response options were "to adopt a new mindset," "diet and nutrition advice," "physical activity advice," "advice to stop clinical care," "to seek treatment outside the US," or "other." Assessed up to 6 times. Assessed at timepoint 4 approximately a week after the day of enrollment.

REFERENCES

1. Peterson JS, Swire-Thompson B, Johnson SB. What is the alternative? Responding strategically to cancer misinformation. *Future Oncology.* 2020;16(25):1883-1888.
2. Warner EL, Waters AR, Cloyes KG, Ellington L, Kirchhoff AC. Young adult cancer caregivers' exposure to cancer misinformation on social media. *Cancer.* 2021;127(8):1318-1324.
3. Vosoughi S, Roy D, Aral S. The spread of true and false news online. *Science.* 2018;359(6380):1146-1151.
4. Chou W-YS, Gaysynsky A, Cappella JN. Where we go from here: health misinformation on social media. *American Journal of Public Health.* 2020;110(53):S273-S275.
5. Chen L, Wang X, Peng T-Q. Nature and diffusion of gynecologic cancer–related misinformation on social media: Analysis of tweets. *Journal of Medical Internet Research.* 2018;20(10):e11515.
6. Loeb S, Reines K, Abu-Salha Y, et al. Quality of bladder cancer information on YouTube. *European Urology.* 2021;79(1):56-59.
7. Gage-Bouchard EA, LaValley S, Warunek M, Beaupin LK, Mollica M. Is cancer information exchanged on social media scientifically accurate? *Journal of Cancer Education.* 2018;33(6):1328-1332.
8. Beeken R, Williams K, Wardle J, Croker H. "What about diet?" A qualitative study of cancer survivors' views on diet and cancer and their sources of information. *European Journal of Cancer Care.* 2016;25(5):774-783.
9. Ghenai A, Mejova Y. Fake cures: user-centric modeling of health misinformation in social media. *Proceedings of the ACM on Human-Computer Interaction.* 2018;2(CSCW):1-20.
10. Johnson SB, Park HS, Gross CP, James BY. Complementary medicine, refusal of conventional cancer therapy, and survival among patients with curable cancers. *JAMA Oncology.* 2018;4(10):1375-1381.
11. Johnson SB, Park HS, Gross CP, Yu JB. Use of alternative medicine for cancer and its impact on survival. *Journal of the National Cancer Institute.* 2018;110(1):121-124.
12. Johnson SB, Parsons M, Dorff T, et al. Cancer misinformation and harmful information on Facebook and other social media: A brief report. *Journal of the National Cancer Institute.* 2022;114(7):1036-1039.
13. Mollica MA, Chou WYS, Tonorezos ES, Smith AW. Young adult caregivers' perceptions of cancer misinformation on social media: Response to Warner et al. *Cancer.* 2021;127(8):1177-1179.
14. Sillence E. Giving and receiving peer advice in an online breast cancer support group. *Cyberpsychology, Behavior, and Social Networking.* 2013;16(6):480-485.
15. *Mobile Fact Sheet* Washington DC: Pew Research Center; April 7, 2021 2021.
16. Perrin A, Atske S. *About three-in-ten U.S. adults say they are 'almost constantly' online.* Pew Research Center; March 26, 2021.
17. Auxier B, Anderson M. *Social media use in 2021.* Pew Research Center; April 7, 2021.
18. Rivera YM, Moran MB, Smith KC. Exploring the role of culture and cognitive heuristics in assessing the credibility of cancer (mis)information on Facebook among U.S. Latinos/as. *Health Communication.* Online first.
19. Friedman B, Pineault K, Nolasco TS, Borno H, Giri V, Loeb S. Quality of information in Spanish on BRCA and genetic testing in prostate cancer on Youtube. *The Journal of Urology.* 2022;207(Supplement 5):e142.
20. Biancovilli P, Jurberg C. How to optimize health messages about cancer on Facebook: Mixed-methods study. *JMIR Cancer.* 2018;4(2):e11073.
21. Soto-Vásquez AD, Gonzalez AA, Shi W, Garcia N, Hernandez J. COVID-19: Contextualizing misinformation flows in a US Latinx border community (media and

communication during COVID-19). *Howard Journal of Communications.* 2021;32(5):421-439.
22. Latané B, Latané B, Darley JM. *The unresponsive bystander: Why doesn't he help?* Appleton-Century-Crofts; 1970.
23. DiFranzo D, Taylor SH, Kazerooni F, Wherry OD, Bazarova NN. Upstanding by design: Bystander intervention in cyberbullying. Proceedings of the 2018 CHI Conference on Human Factors in Computing Systems. 2018.
24. Taylor SH, DiFranzo D, Choi YH, Sannon S, Bazarova NN. Accountability and empathy by design: Encouraging bystander intervention to cyberbullying on social media. *Proceedings of the ACM on Human-Computer Interaction.* 2019;3(CSCW):1-26.
25. Wang S. Standing up or standing by: Bystander intervention in cyberbullying on social media. *New Media & Society.* 2021;23(6):1379-1397.
26. Dillon KP, Bushman BJ. Unresponsive or un-noticed?: Cyberbystander intervention in an experimental cyberbullying context. *Computers in Human Behavior.* 2015;45:144-150.
27. Perez S. Twitter expands misinformation reporting feature to more international markets. Tech Crunch. https://techcrunch.com/2022/01/18/twitter-expands-misinformation-reporting-feature-to-more-international-markets. January 18, 2022.
28. Coleman K. Introducing Birdwatch, a community-based approach to misinformation. Twitter. Published 2021. Updated January 25, 2021. Accessed.
29. Matney L. Twitter starts rolling out Birdwatch fact checks inside tweets. Tech Crunch. https://techcrunch.com/2021/06/02/twitter-starts-rolling-out-birdwatch-fact-checks-inside-tweets/. June 2, 2021.
30. Vraga EK, Bode L. Correction as a solution for health misinformation on social media. In. Vol 110: American Public Health Association; 2020:S278-S280.
31. Walter N, Brooks JJ, Saucier CJ, Suresh S. Evaluating the impact of attempts to correct health misinformation on social media: A meta-analysis. *Health Communication.* 2021;36(13):1776-1784.
32. Bode L, Vraga EK. See something, say something: Correction of global health misinformation on social media. *Health Communication.* 2018;33(9):1131-1140.

STATISICAL ANALYSIS PLAN

Social media posts about cancer:
A randomized controlled trial of user interface designs to encourage intervening and reduce sharing of cancer treatment misinformation

Principal Investigator
Allison J. Lazard, PhD

Biostatistician
Tara Licciardello Queen, PhD

Study site. University of North Carolina at Chapel Hill

Version. 6.26.2026

PROTOCOL SYNOPSIS

**Study title**	Social media posts about cancer
**Sponsor**	University of North Carolina at Chapel Hill (UNC) Lineberger Comprehensive Cancer Center
**Study rationale**	Cancer misinformation exerts a heavy burden on cancer patients, survivors, and caregivers.<br>Cancer misinformation about treatment and cures is often spread by family and friends.<br>Strategies to encourage intervening with cancer misinformation and prevent sharing are needed.<br>Social media user interface designs, including in-platform prompts and policies, have promise to reduce cancer misinformation by encouraging pro-social intervening.
**Study objectives**	Primary. To examine whether user interface designs (social cue prompts and policy, standard prompts) lead to more intervening of cancer misinformation.<br>Secondary. To examine whether user interface designs lead to less sharing over time and whether the impact of prompts change over time.<br>Exploratory. To explore whether user interface designs influence intervening outside of the study social media platform. To descriptively contextualize primary and secondary objective finding with behavior and reactions for cancer support posts.
**Study design**	Parallel randomized controlled trial (RCT)
**Number of participants**	294 participants
**Study duration**	Each participant was in the trial for ~2 weeks. The trial was open from March 31 to May 12, 2025.
**Study phases**	Participants were invited to complete 5 surveys. Each survey was open 2 business days apart.<br>Participants were randomized to trial arm in the first survey (Time 1).<br>Participants saw cancer misinformation posts with social cue-enhanced prompts that specify others' action and a review policy (experimental: social cue prompts and policy), cancer misinformation posts with standard prompts (experimental: standard prompts), or cancer misinformation posts with no prompts (control) throughout the RCT.<br>In each survey (Times 1-5), participants scrolled through posts on our stimulated social media platform, *Invibe,* to view the stimuli (passive exposure). Participants then saw the stimuli posts on following webpages in the survey (forced exposure) and responded to self-report items.

STATISTICAL ANALYSIS PLAN

Predictions

We hypothesized that social cue prompts and standard prompts will lead to more prosocial intervening compared to the no-prompt control (Hypothesis 1). We hypothesized that social cue

prompts will lead to more prosocial intervening compared to standard prompts (Hypothesis 2). We asked whether the impact of social cue or standard prompts change over time. We also asked whether prompts (social cue or standard) impact secondary outcomes compared to the control. We explored whether the prompts (social cue or standard) influenced how participants intervened with cancer misinformation in their lives (i.e., outside of the study platform).

Statistical Methods

Analyses were intent-to-treat, including all participants randomized at Time 1. We used a critical alpha of .05 or 95% confidence intervals, and two-tailed tests to conduct all statistical analyses.

We descriptively present demographics overall and by trial arm. We did not test trial arm differences at baseline, following CONSORT guidelines.

All behavioral outcomes (flagging, disliking, liking) were constrained to posts with exposure.

All behavioral and self-reported outcomes related to social media posts were analyzed using generalized estimating equations (GEE) for repeated measures.

For the dichotomous behavioral outcome models, we used counterfactuals and weighting to help with convergence. We padded the dataset with counterfactuals and weighted original observations as high (e.g, 0.9999) and low (e.g., 0.00001). For each response in the dataset, we placed a counterfactual (1-var).

Primary outcomes. To examine the impact of trial arm on the primary outcome, we estimated two models using GEE. First, we analyzed flagging behavior. Flagging was treated as a binary outcome (1 = post flagged, 0 = post not flagged), and each participant had up to 10 observations (i.e., 2 posts at each timepoint, Times 1-5). Predictor variables included the three-level categorical indicator for trial arm (between persons), a five-level categorical indicator of time (within persons), and an interaction between the two. We reported rate ratios and confidence intervals for each trial arm contrast. The trial arm contrasts and p-values reported are from the model that involves trial arm, time, and the interaction of the two. Analysis treated the intercept as random.

Second, we analyzed self-reported willingness to flag using GEE. Willingness to flag was treated as a linear outcome, and each participant had up to 10 observations (i.e., 2 posts at each timepoint, Times 1-5). Predictor variables included the three-level categorical indicator for trial arm (between persons), a five-level categorical indicator of time (within persons), and an interaction between the two. We reported mean differences and confidence intervals for each trial arm contrast. Analysis treated the intercept as random.

Secondary outcomes. We examined our secondary behavioral outcomes of whether participants disliked or liked a post with the same process as flagging (dichotomous). Disliking behavior and liking behavior were each modeled in GEE with the same set of predictors including fixed effects of trial arm (between subjects, 3 levels), trial timepoint (within subjects, 5 levels), and an interaction between the two.

We examined our secondary self-reported outcomes of willingness to dislike, willingness to like, willingness to comment, and willingness to share in the same way as willingness to flag (linear). Each continuous outcome was modeled in GEE, separately, with the same set of predictors including fixed effects of trial arm (between subjects, 3-level categorical indicator), trial timepoint (within subjects, 5-level categorical indicator), and the interaction between the two.

We descriptively reported the frequencies of participants' self-reported comment valence (e.g., agree, disagree), sharing recipients, and sharing channels. For each outcome, we recoded responses for each participant into dichotomous indicators representing whether participants made any selection during the trial for each response option (e.g., 0 = not selected during the trial; 1 = selected response for one or more posts during the trial). Frequencies of these binary indicators were then calculated to summarize the proportion of participants endorsing each outcome by trial arm.

Exploratory outcomes. To assess whether there were differences by trial arm for intervening with social media posts outside of the simulated platform in the past week, we conducted logistic regressions. Outcomes in these models included: disliked posts, flagged posts, muted accounts, commented to correct posts, chatted with others to disagree, or encouraged someone to flag posts. We conducted one model for each intervening outcome (7 outcomes total) with trial arm (between subjects, 3 level-categorical indicator) as the predictor We descriptively reported participants' self-report social media platforms where intervening occurred, intervening recipients, and topics of cancer misinformation.

Sample size

To estimate the sample size needs, we conducted an *a priori* power analysis for our continuous outcome of willingness. We assumed three trial arms of equal size (1:1:1), five repeated measures, an interclass correlation coefficient of .67 (based on pilot data), and a *Cohen's f* effect size of .20, for a two-sided $\alpha = 0.05$ with 80% power. The effect size estimate was based on pilot findings on differences between willingness to flag after exposure to social cue prompts or standard prompts (between "somewhat willing" M = 2.99, SD = 1.37 and "slightly willing" M = 2.34, SD = 1.39 at Time 1) in the simulated social media environment.[1] We also conducted power calculations for a social media flagging behavior, a binary outcome. Estimates were informed by pilot data from the same prior intervention-only study, which demonstrated event rates of 6% and 16% across comparison conditions at Time 1.[1] Assuming a two-sided $\alpha = 0.05$, 80% power, and an intraclass correlation coefficient (ICC) of 0.50 to account for within-cluster correlation, the required total sample size was 123 participants. With these parameters, we aimed to collect data from 300 participants, assuming 70% retention or greater, to have a sample of at least 222 individuals who completed 3 or more timepoints.

Reference

1. Lazard AJ, Queen TL, Pulido M, Lake S, Nicolla S, Tan HJ, Charlot M, Smitherman AB, Dasgupta N. Social media prompts to encourage intervening with cancer treatment misinformation. *Social Science & Medicine.* 2025;372:117950.

Cancer misinformation posts, **Social cue condition**

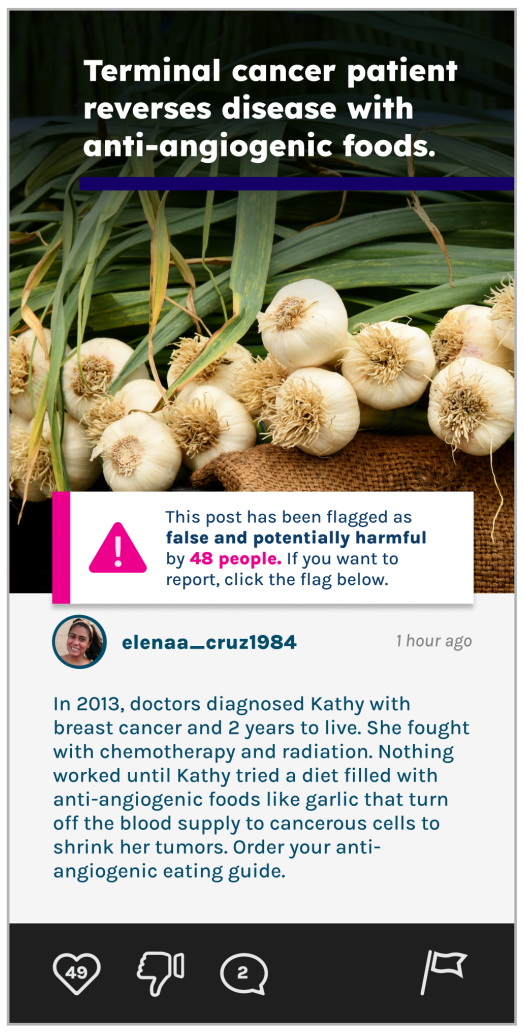

Time 1, post 1

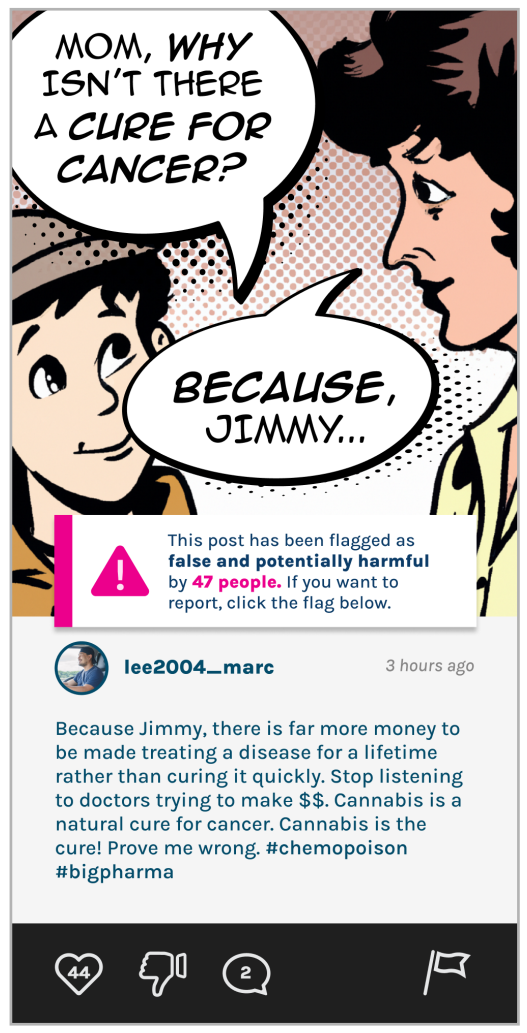

Time 1, post 2

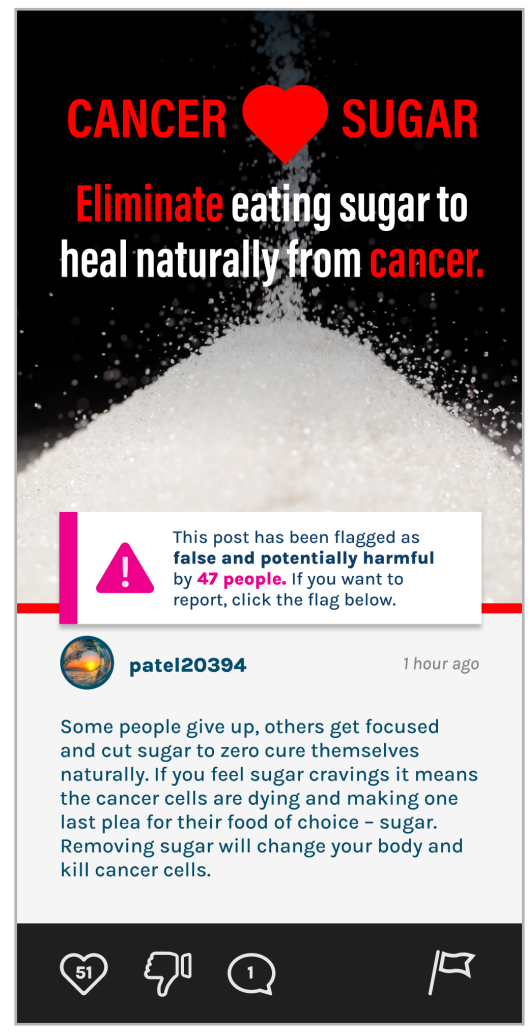

Time 2, post 1

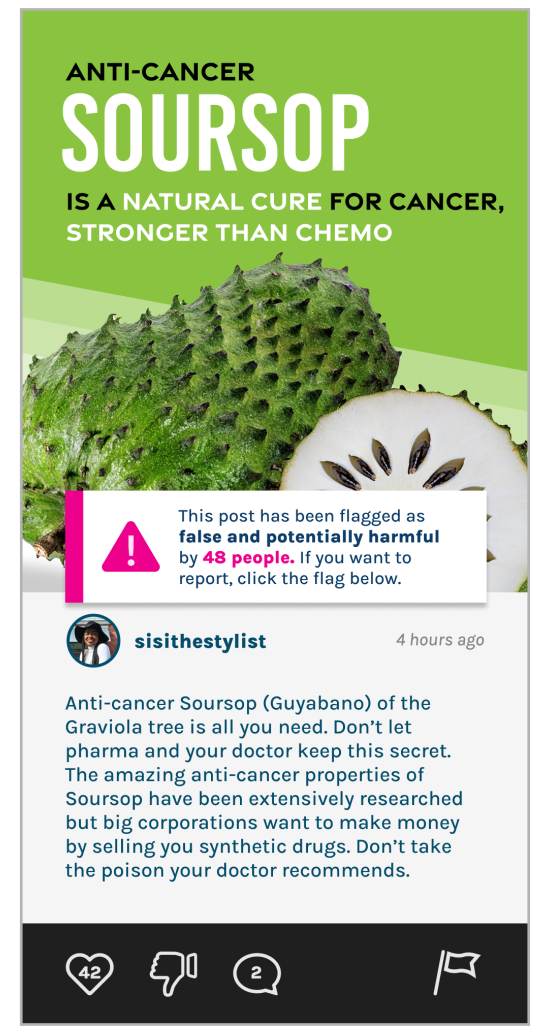

Time 2, post 2

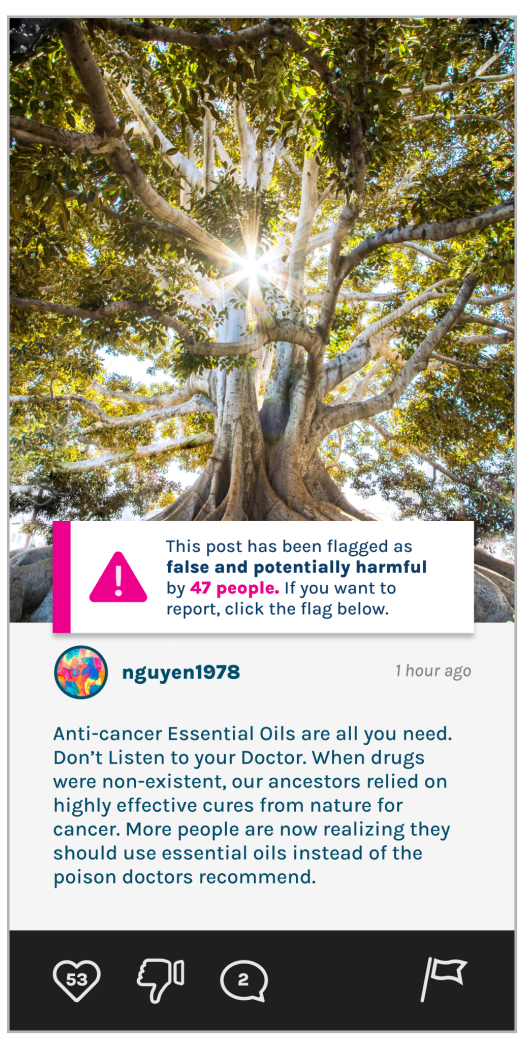

Time 3, post 1

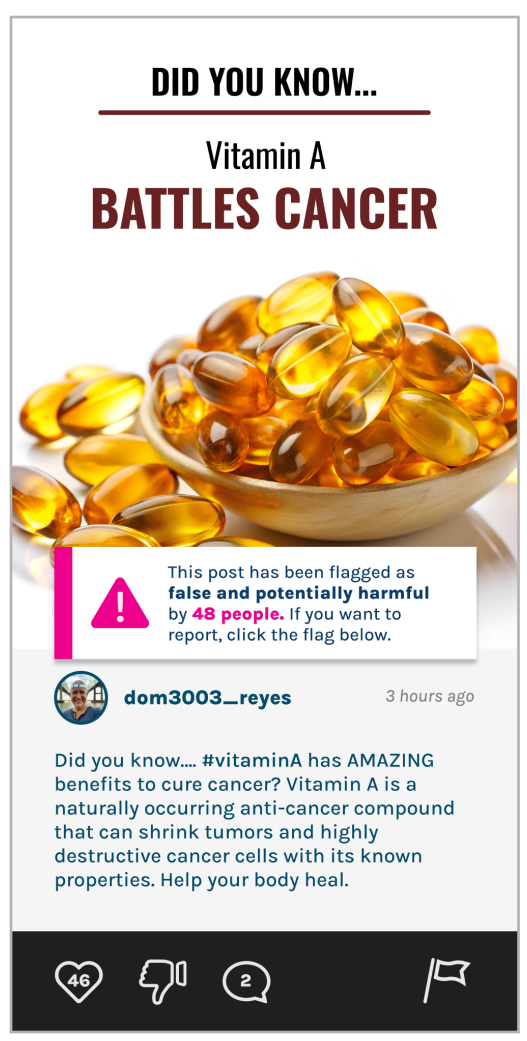

Time 3, post 2

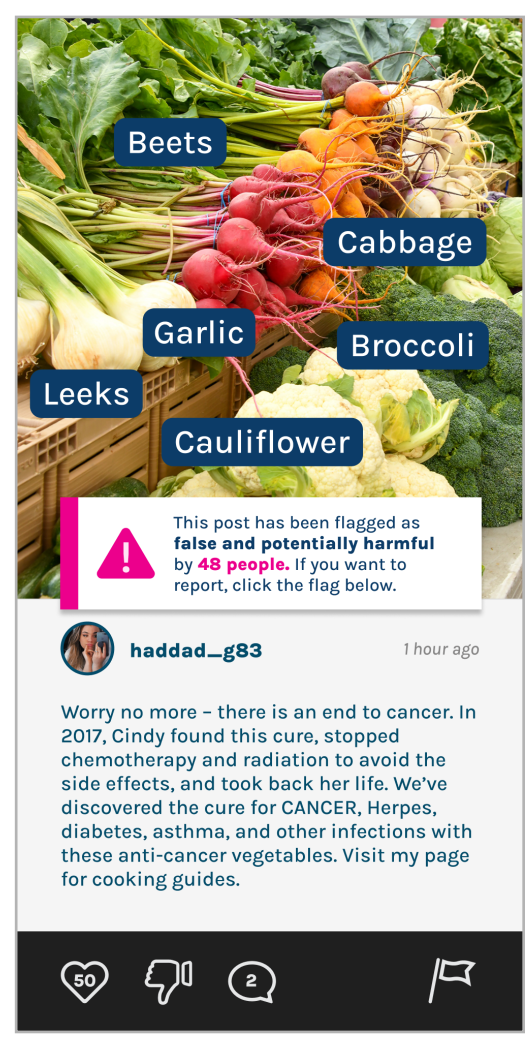

Time 4, post 1

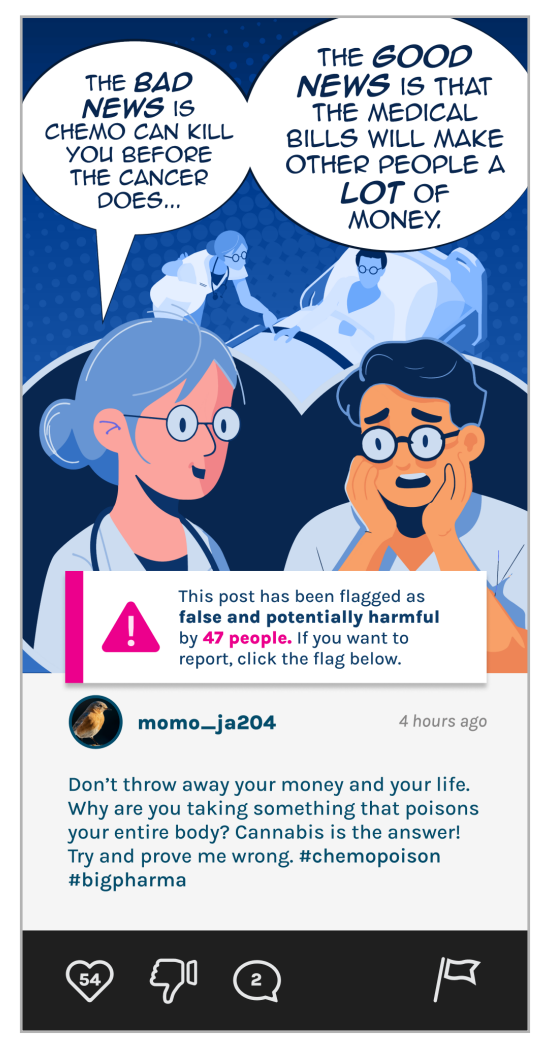

Time 4, post 2

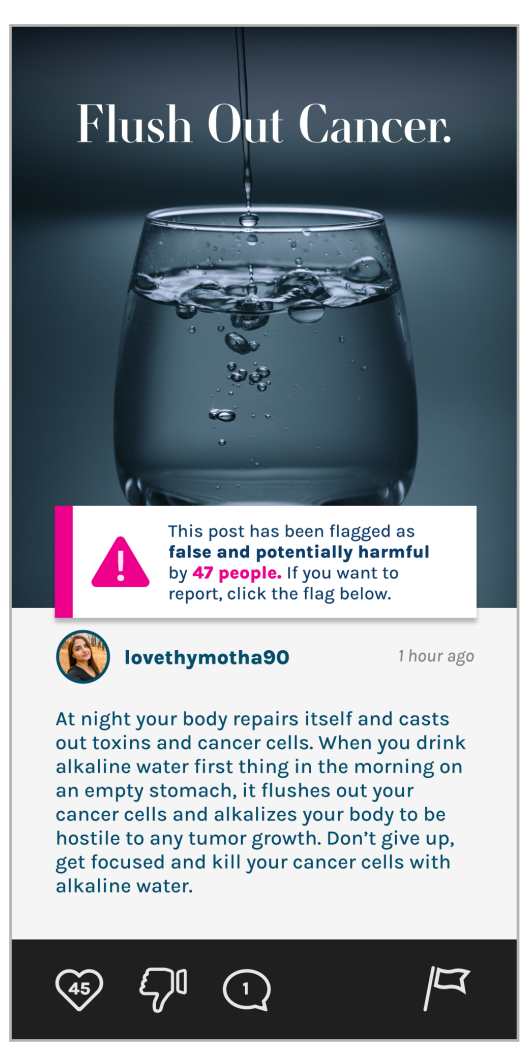

Time 5, post 1

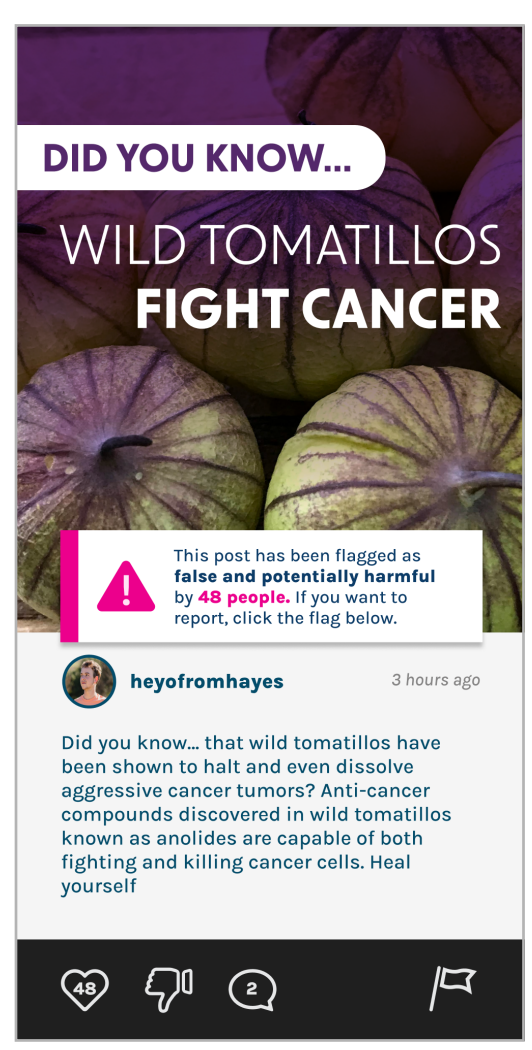

Time 5, post 2

Cancer misinformation posts, **Standard condition**

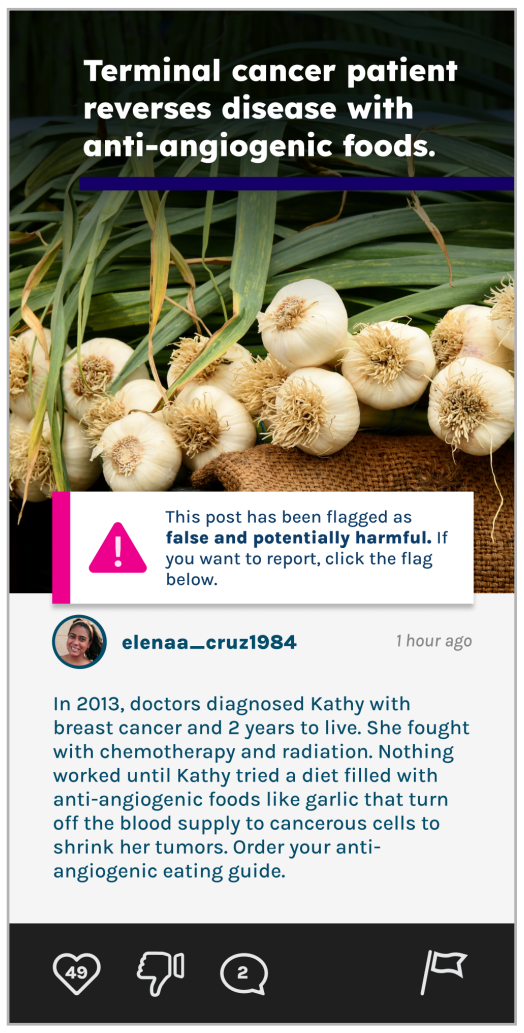

Time 1, post 1

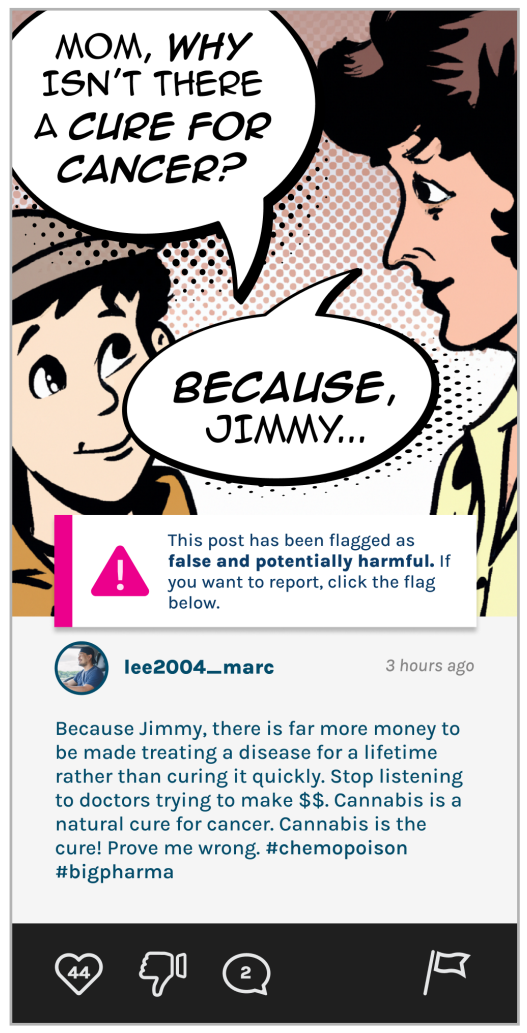

Time 1, post 2

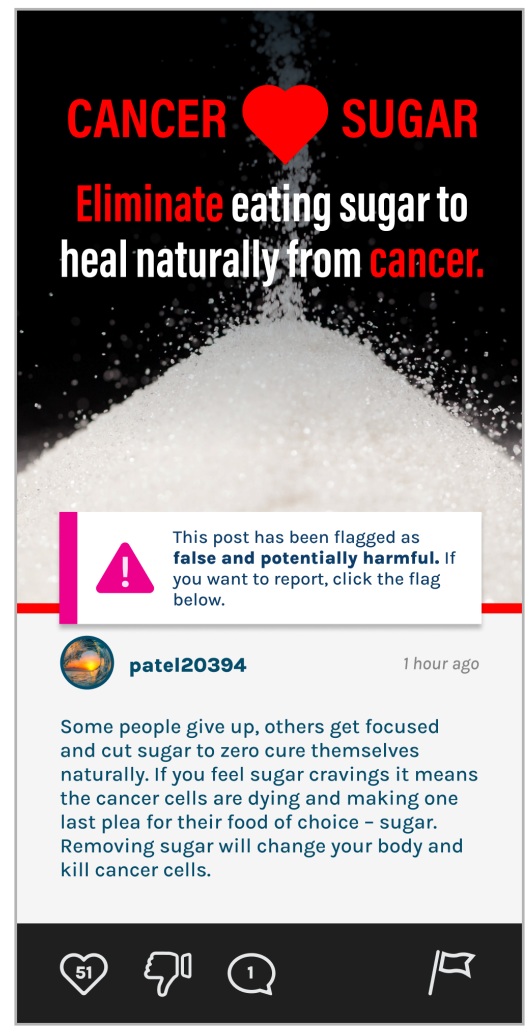

Time 2, post 1

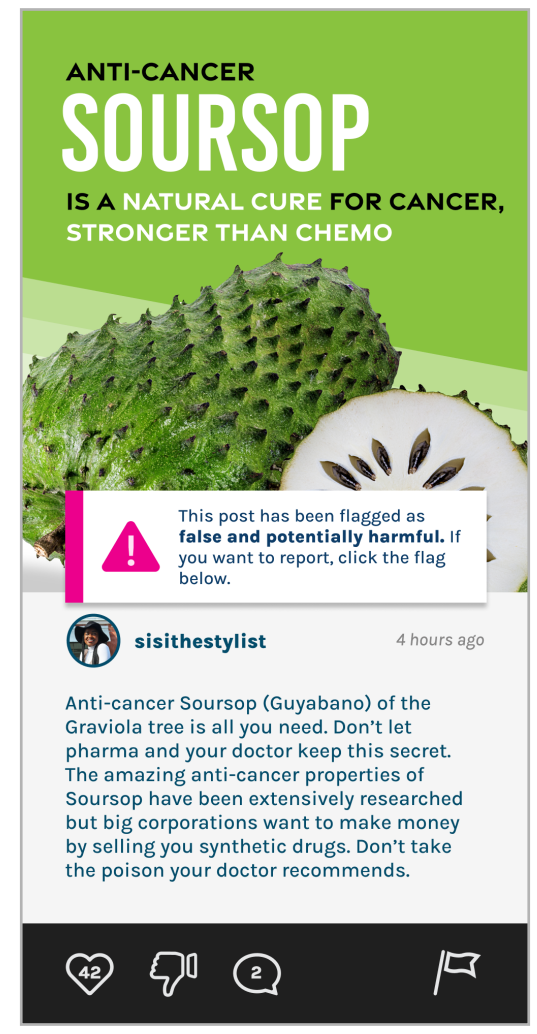

Time 2, post 2

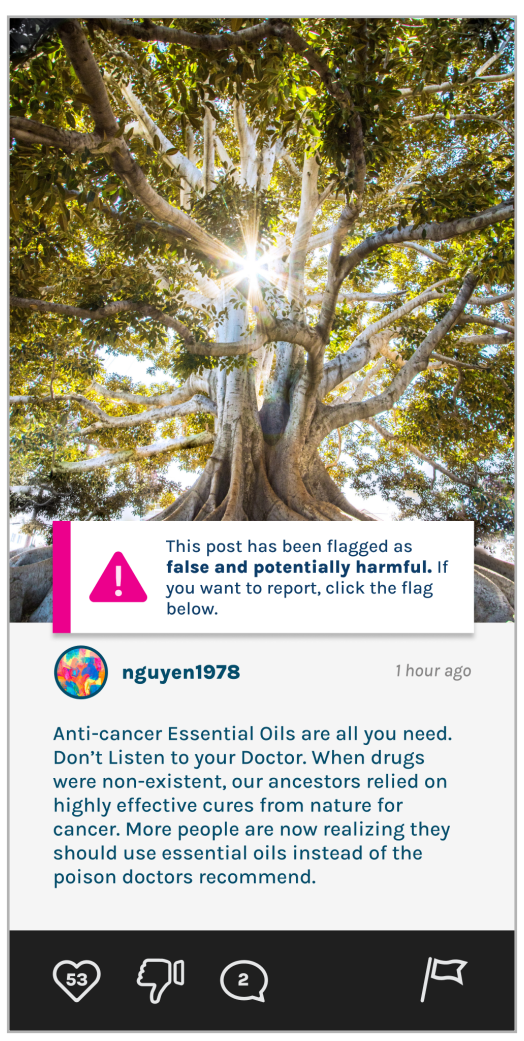

Time 3, post 1

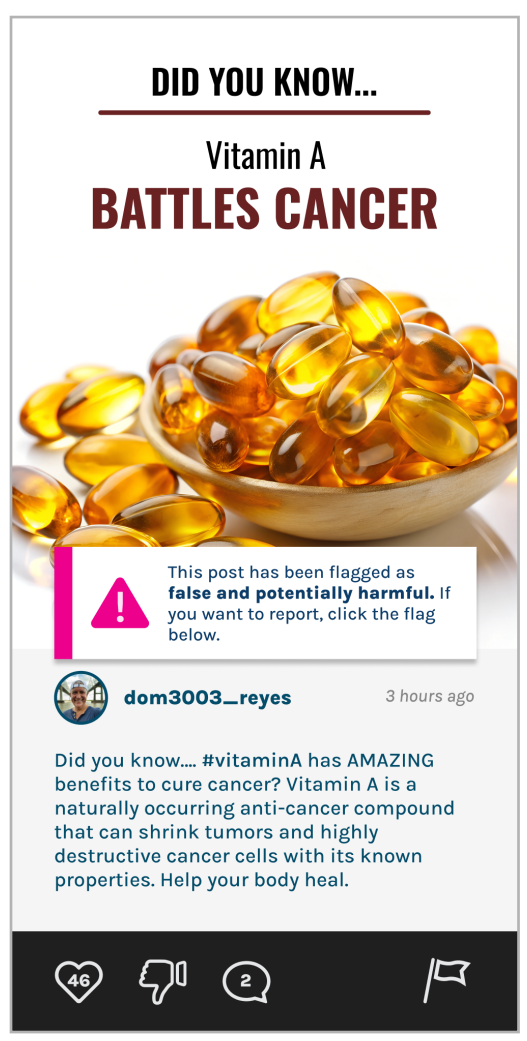

Time 3, post 2

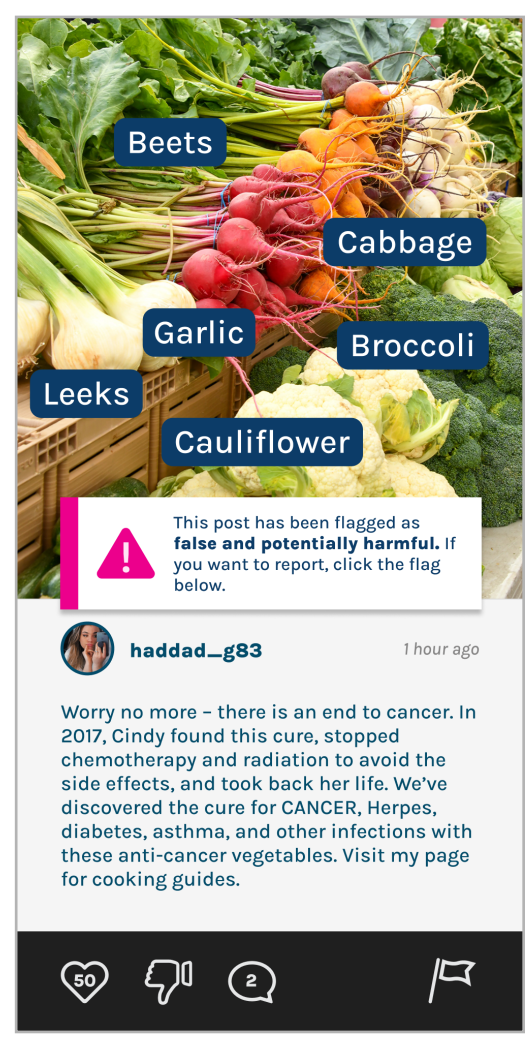

Time 4, post 1

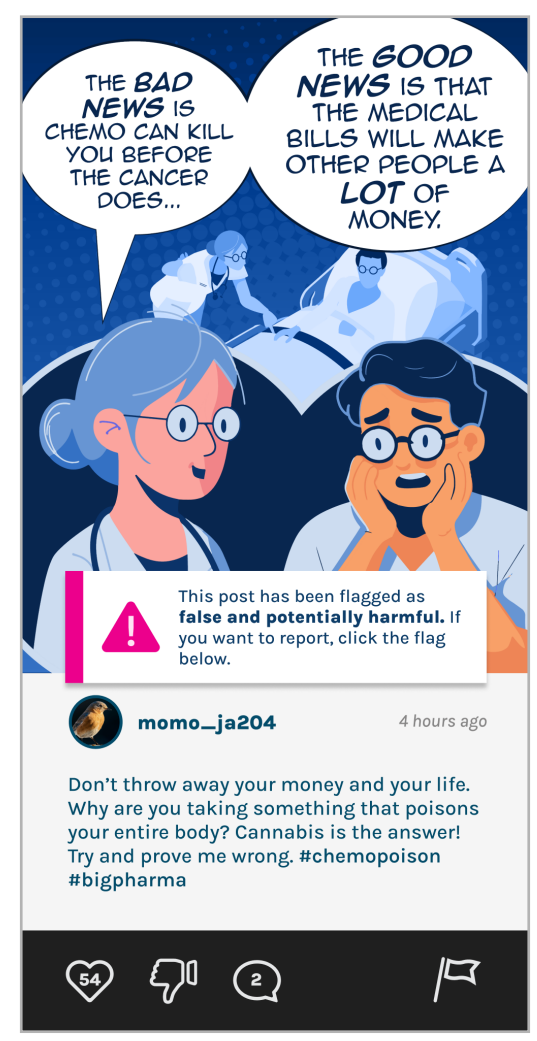

Time 4, post 2

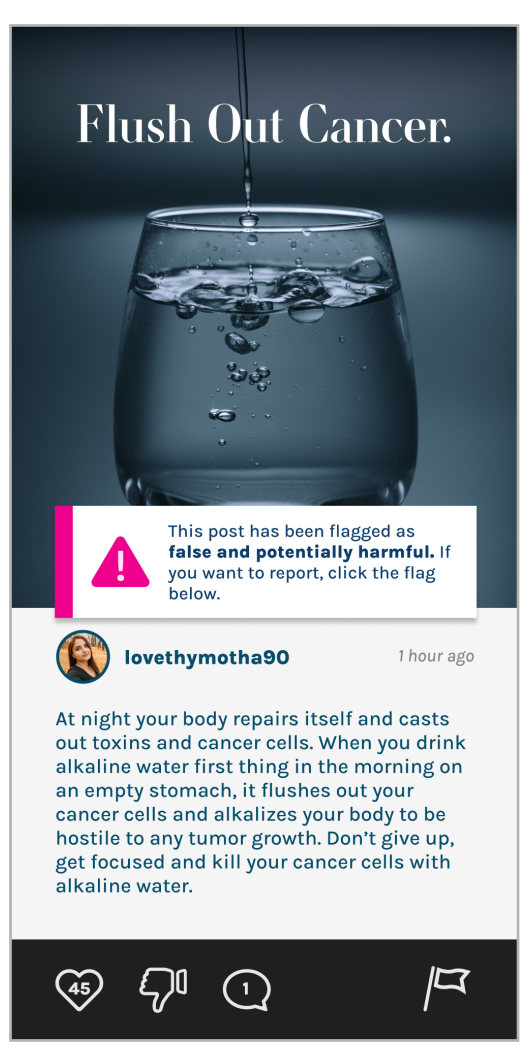

Time 5, post 1

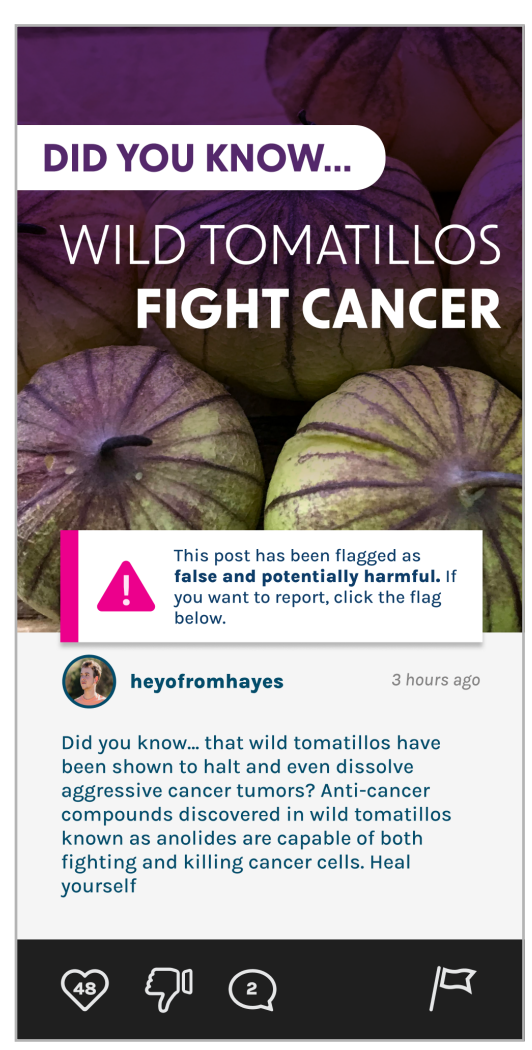

Time 5, post 2

Cancer misinformation posts, **Control condition**

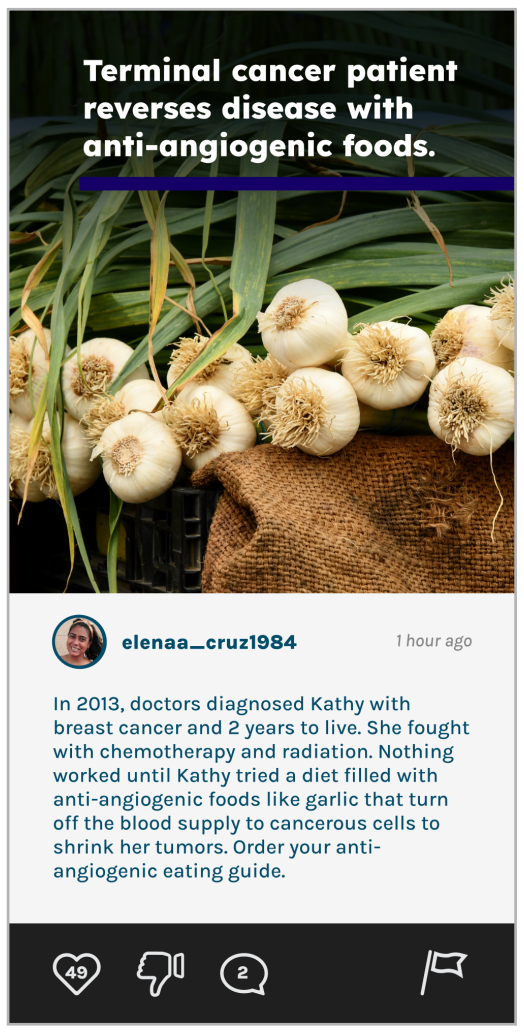

Time 1, post 1

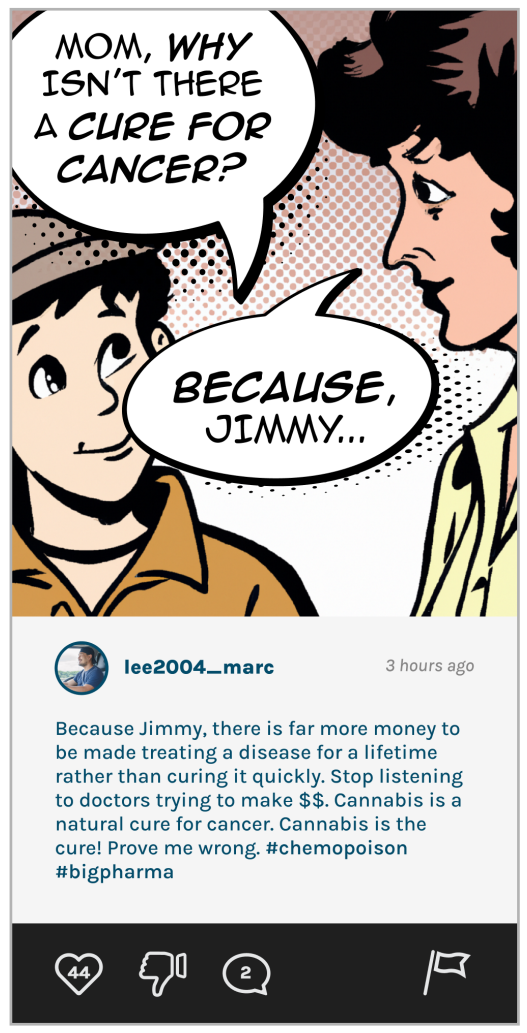

Time 1, post 2

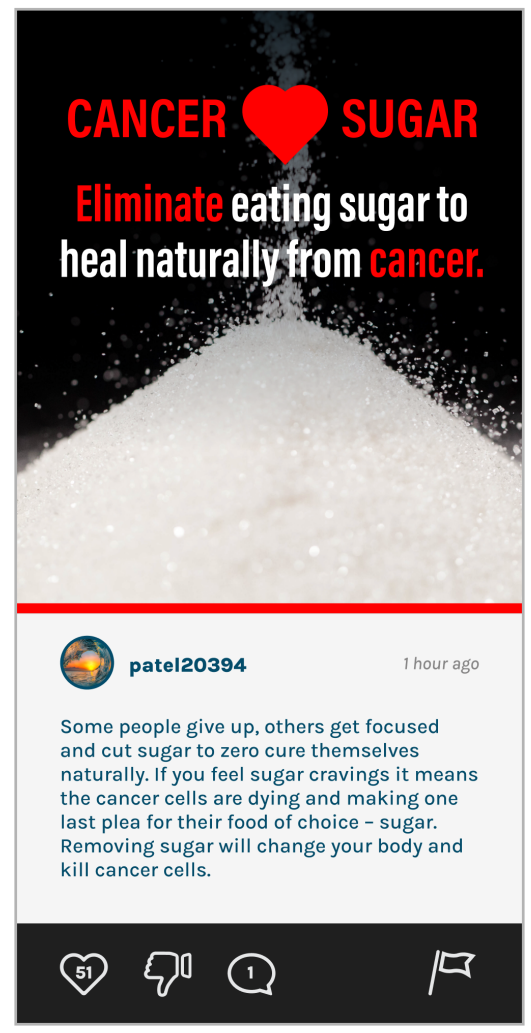

Time 2, post 1

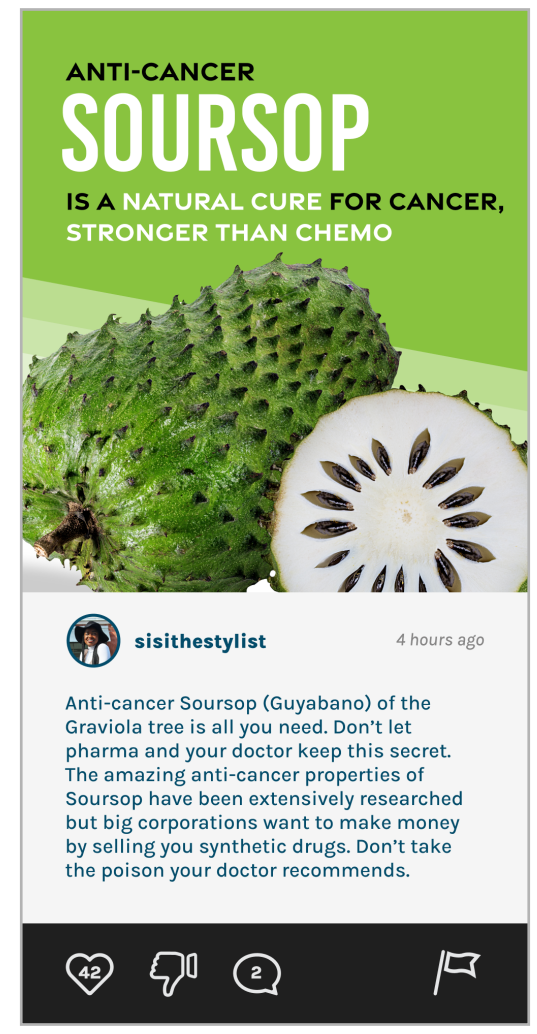

Time 2, post 2

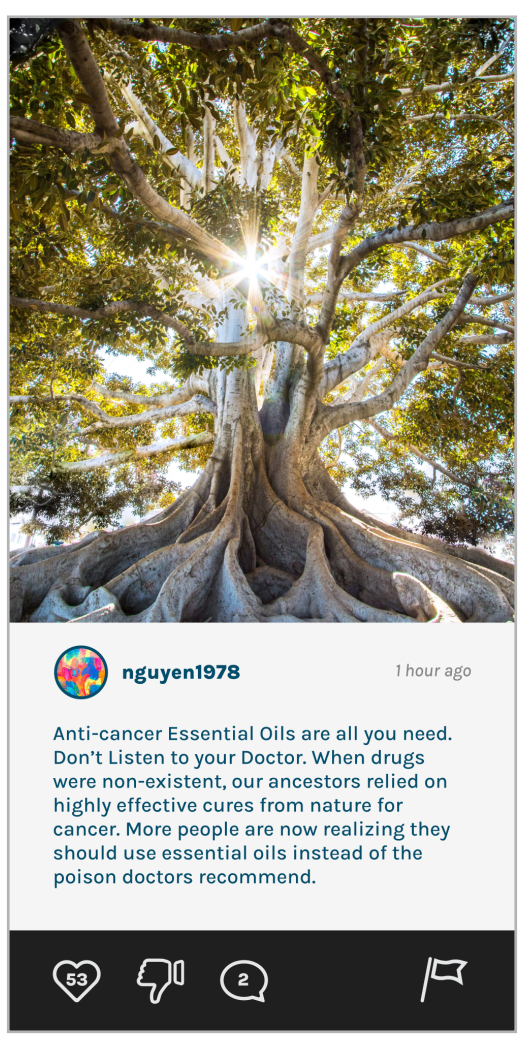

Time 3, post 1

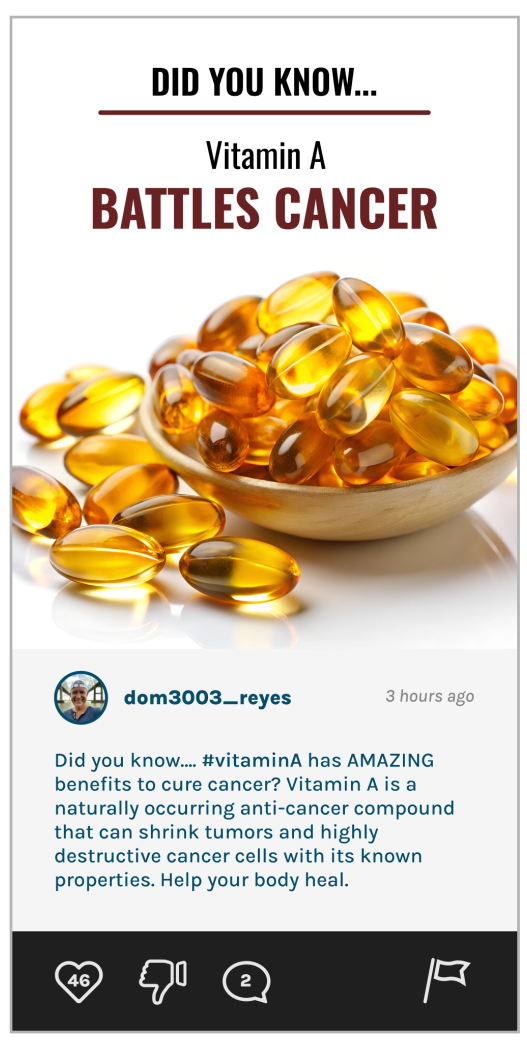

Time 3, post 2

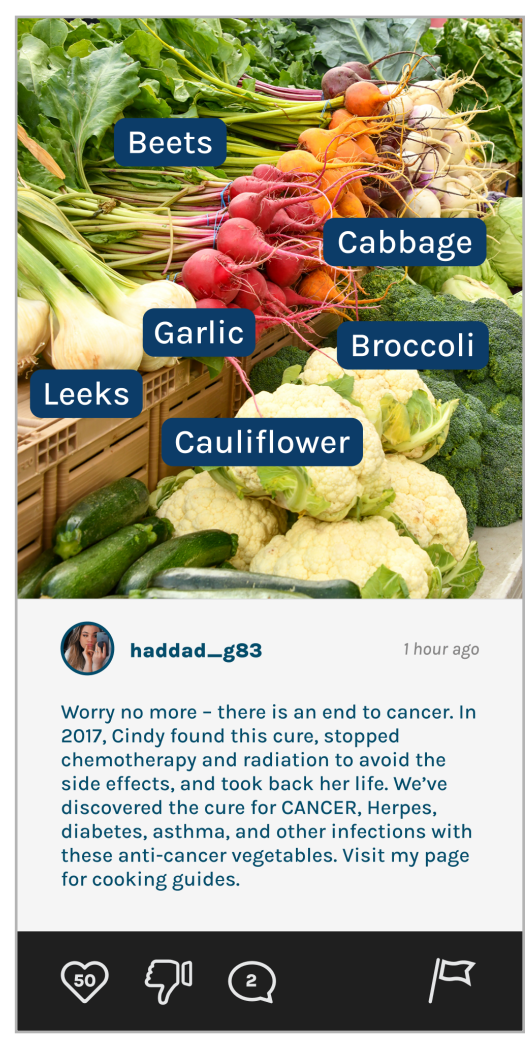

Time 4, post 1

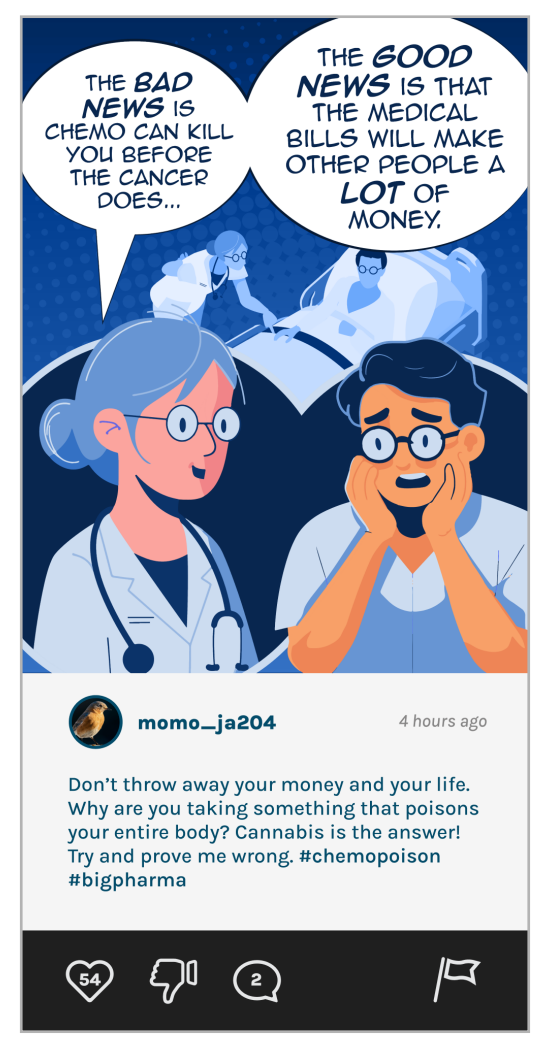

Time 4, post 2

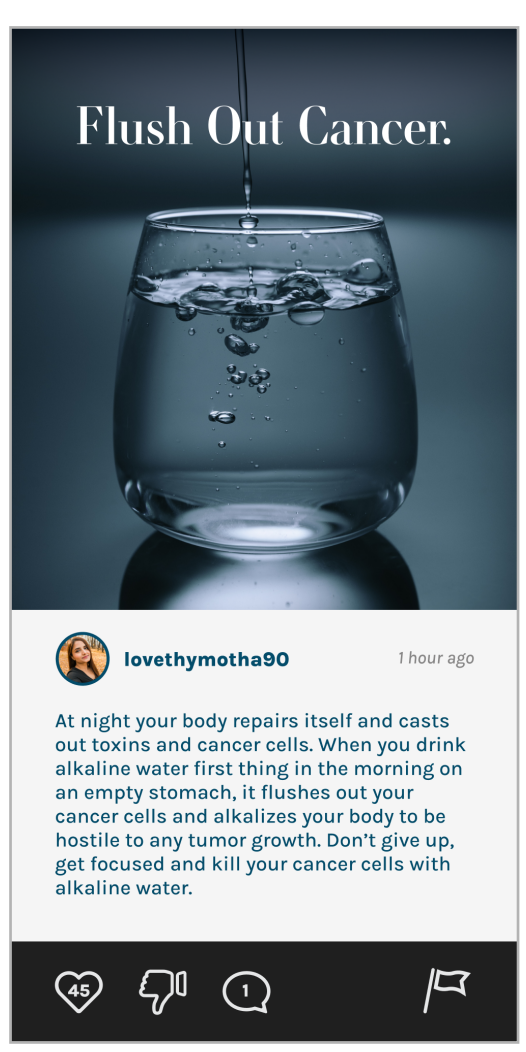

Time 5, post 1

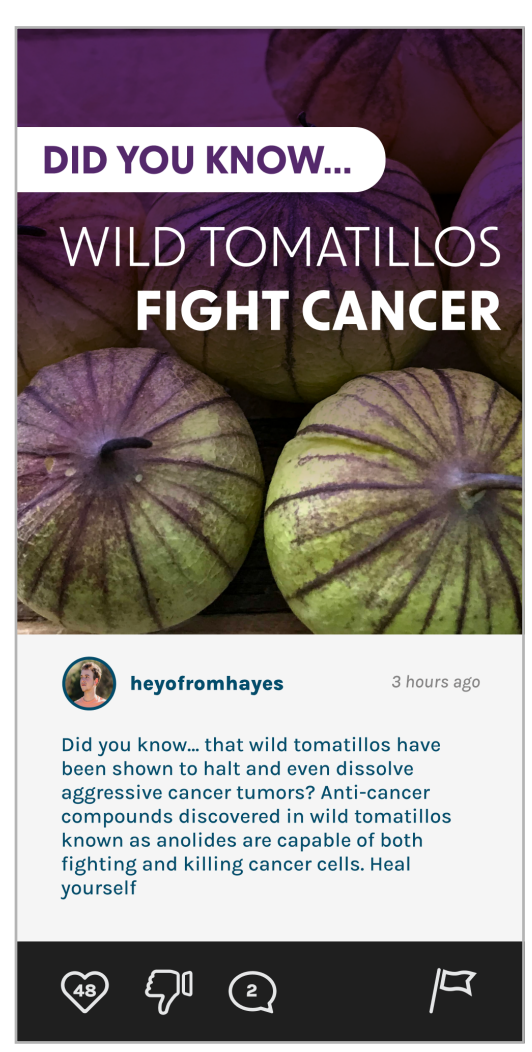

Time 5, post 2

Cancer support posts

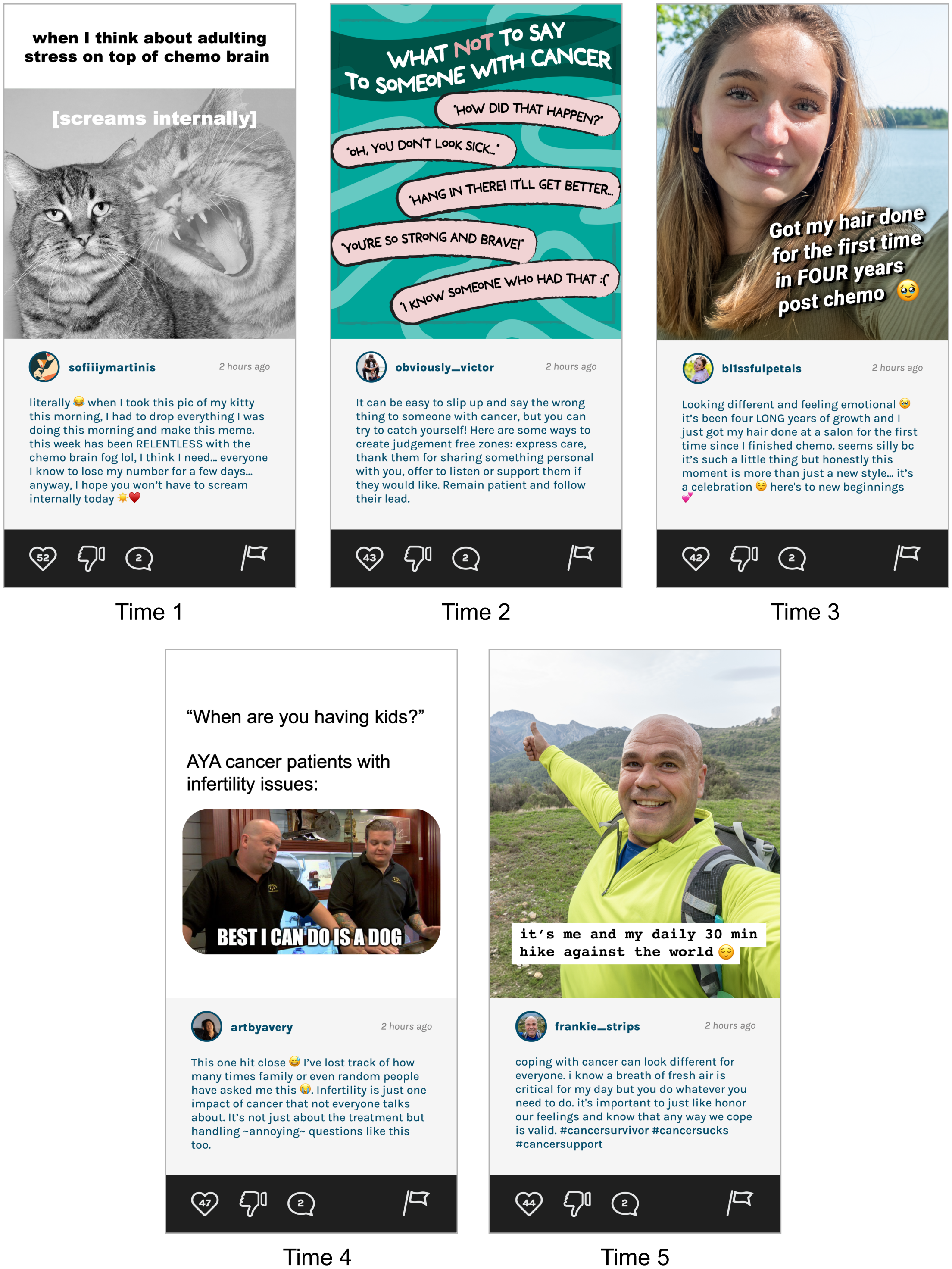

Example background posts (non-stimuli)

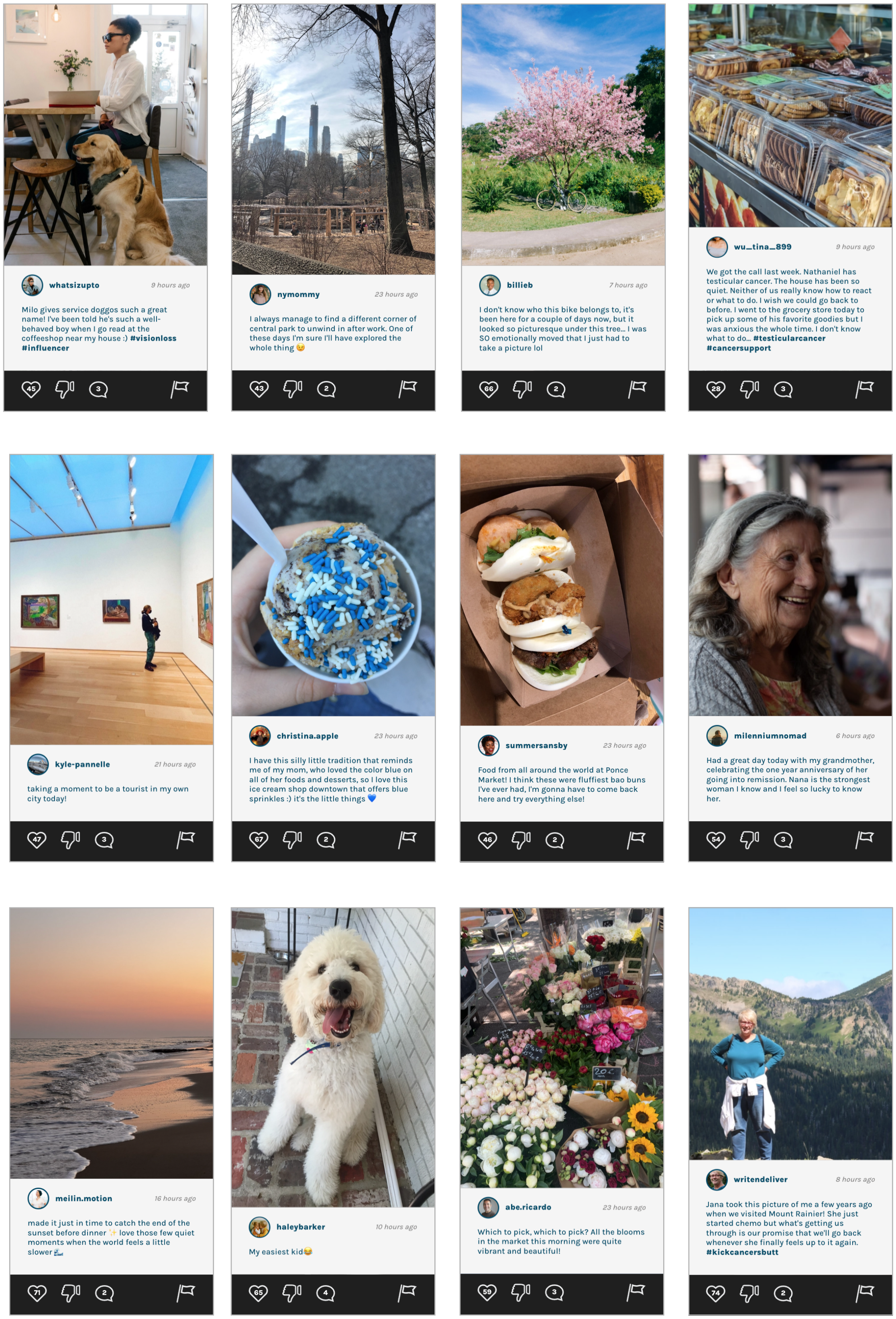

STATISICAL ANALYSIS PLAN

Social media posts about cancer:

A randomized controlled trial of user interface designs to encourage intervening and reduce sharing of cancer treatment misinformation

Principal Investigator
Allison J. Lazard, PhD

Biostatistician
Tara Licciardello Queen, PhD

Study site. University of North Carolina at Chapel Hill

Version. 6.26.2026

PROTOCOL SYNOPSIS

**Study title**	Social media posts about cancer
**Sponsor**	University of North Carolina at Chapel Hill (UNC) Lineberger Comprehensive Cancer Center
**Study rationale**	Cancer misinformation exerts a heavy burden on cancer patients, survivors, and caregivers.<br>Cancer misinformation about treatment and cures is often spread by family and friends.<br>Strategies to encourage intervening with cancer misinformation and prevent sharing are needed.<br>Social media user interface designs, including in-platform prompts and policies, have promise to reduce cancer misinformation by encouraging pro-social intervening.
**Study objectives**	Primary. To examine whether user interface designs (social cue prompts and policy, standard prompts) lead to more intervening of cancer misinformation.<br>Secondary. To examine whether user interface designs lead to less sharing over time and whether the impact of prompts change over time.<br>Exploratory. To explore whether user interface designs influence intervening outside of the study social media platform. To descriptively contextualize primary and secondary objective finding with behavior and reactions for cancer support posts.
**Study design**	Parallel randomized controlled trial (RCT)
**Number of participants**	294 participants
**Study duration**	Each participant was in the trial for ~2 weeks. The trial was open from March 31 to May 12, 2025.
**Study phases**	Participants were invited to complete 5 surveys. Each survey was open 2 business days apart.<br>Participants were randomized to trial arm in the first survey (Time 1).<br>Participants saw cancer misinformation posts with social cue-enhanced prompts that specify others' action and a review policy (experimental: social cue prompts and policy), cancer misinformation posts with standard prompts (experimental: standard prompts), or cancer misinformation posts with no prompts (control) throughout the RCT.<br>In each survey (Times 1-5), participants scrolled through posts on our stimulated social media platform, *Invibe,* to view the stimuli (passive exposure). Participants then saw the stimuli posts on following webpages in the survey (forced exposure) and responded to self-report items.

STATISTICAL ANALYSIS PLAN

Predictions

We hypothesized that social cue prompts and standard prompts will lead to more prosocial intervening compared to the no-prompt control (Hypothesis 1). We hypothesized that social cue

prompts will lead to more prosocial intervening compared to standard prompts (Hypothesis 2). We asked whether the impact of social cue or standard prompts change over time. We also asked whether prompts (social cue or standard) impact secondary outcomes compared to the control. We explored whether the prompts (social cue or standard) influenced how participants intervened with cancer misinformation in their lives (i.e., outside of the study platform).

Statistical Methods

Analyses were intent-to-treat, including all participants randomized at Time 1. We used a critical alpha of .05 or 95% confidence intervals, and two-tailed tests to conduct all statistical analyses.

We descriptively present demographics overall and by trial arm. We did not test trial arm differences at baseline, following CONSORT guidelines.

All behavioral outcomes (flagging, disliking, liking) were constrained to posts with exposure.

All behavioral and self-reported outcomes related to social media posts were analyzed using generalized estimating equations (GEE) for repeated measures.

For the dichotomous behavioral outcome models, we used counterfactuals and weighting to help with convergence. We padded the dataset with counterfactuals and weighted original observations as high (e.g, 0.9999) and low (e.g., 0.00001). For each response in the dataset, we placed a counterfactual (1-var).

Primary outcomes. To examine the impact of trial arm on the primary outcome, we estimated two models using GEE. First, we analyzed flagging behavior. Flagging was treated as a binary outcome (1 = post flagged, 0 = post not flagged), and each participant had up to 10 observations (i.e., 2 posts at each timepoint, Times 1-5). Predictor variables included the three-level categorical indicator for trial arm (between persons), a five-level categorical indicator of time (within persons), and an interaction between the two. We reported rate ratios and confidence intervals for each trial arm contrast. The trial arm contrasts and p-values reported are from the model that involves trial arm, time, and the interaction of the two. Analysis treated the intercept as random.

Second, we analyzed self-reported willingness to flag using GEE. Willingness to flag was treated as a linear outcome, and each participant had up to 10 observations (i.e., 2 posts at each timepoint, Times 1-5). Predictor variables included the three-level categorical indicator for trial arm (between persons), a five-level categorical indicator of time (within persons), and an interaction between the two. We reported mean differences and confidence intervals for each trial arm contrast. Analysis treated the intercept as random.

Secondary outcomes. We examined our secondary behavioral outcomes of whether participants disliked or liked a post with the same process as flagging (dichotomous). Disliking behavior and liking behavior were each modeled in GEE with the same set of predictors including fixed effects of trial arm (between subjects, 3 levels), trial timepoint (within subjects, 5 levels), and an interaction between the two.

We examined our secondary self-reported outcomes of willingness to dislike, willingness to like, willingness to comment, and willingness to share in the same way as willingness to flag (linear). Each continuous outcome was modeled in GEE, separately, with the same set of predictors including fixed effects of trial arm (between subjects, 3-level categorical indicator), trial timepoint (within subjects, 5-level categorical indicator), and the interaction between the two.

We descriptively reported the frequencies of participants' self-reported comment valence (e.g., agree, disagree), sharing recipients, and sharing channels. For each outcome, we recoded responses for each participant into dichotomous indicators representing whether participants made any selection during the trial for each response option (e.g., 0 = not selected during the trial; 1 = selected response for one or more posts during the trial). Frequencies of these binary indicators were then calculated to summarize the proportion of participants endorsing each outcome by trial arm.

Exploratory outcomes. To assess whether there were differences by trial arm for intervening with social media posts outside of the simulated platform in the past week, we conducted logistic regressions. Outcomes in these models included: disliked posts, flagged posts, muted accounts, commented to correct posts, chatted with others to disagree, or encouraged someone to flag posts. We conducted one model for each intervening outcome (7 outcomes total) with trial arm (between subjects, 3 level-categorical indicator) as the predictor We descriptively reported participants' self-report social media platforms where intervening occurred, intervening recipients, and topics of cancer misinformation.

Sample size

To estimate the sample size needs, we conducted an *a priori* power analysis for our continuous outcome of willingness. We assumed three trial arms of equal size (1:1:1), five repeated measures, an interclass correlation coefficient of .67 (based on pilot data), and a *Cohen's f* effect size of .20, for a two-sided α = 0.05 with 80% power. The effect size estimate was based on pilot findings on differences between willingness to flag after exposure to social cue prompts or standard prompts (between "somewhat willing" M = 2.99, SD = 1.37 and "slightly willing" M = 2.34, SD = 1.39 at Time 1) in the simulated social media environment.[1] We also conducted power calculations for a social media flagging behavior, a binary outcome. Estimates were informed by pilot data from the same prior intervention-only study, which demonstrated event rates of 6% and 16% across comparison conditions at Time 1.[1] Assuming a two-sided α = 0.05, 80% power, and an intraclass correlation coefficient (ICC) of 0.50 to account for within-cluster correlation, the required total sample size was 123 participants. With these parameters, we aimed to collect data from 300 participants, assuming 70% retention or greater, to have a sample of at least 222 individuals who completed 3 or more timepoints.

Reference

1. Lazard AJ, Queen TL, Pulido M, Lake S, Nicolla S, Tan HJ, Charlot M, Smitherman AB, Dasgupta N. Social media prompts to encourage intervening with cancer treatment misinformation. *Social Science & Medicine.* 2025;372:117950.
