# Supplementary material for: User Interface Designs to Address Cancer Misinformation: A Randomized Clinical Trial
Source: JAMA Netw Open. 2026 Oct 29;9(10):e2641122. doi: 10.1001/jamanetworkopen.2026.41122 (PMC13720694; doi:10.1001/jamanetworkopen.2026.41122)
Supplement: Supplementary file 2 [file jamanetwopen-e2641122-s002.pdf]

Supplemental Online Content

Lazard AJ, Avendaño-Galdamez M, Queen TL, et al. User interface designs to address cancer misinformation: a randomized clinical trial. *JAMA Netw Open.* 2026;9(10):e2641122. doi:10.1001/jamanetworkopen.2026.41122

eTable 1. Survey items for self-reported willingness to intervene and share

eTable 2. Model means and condition comparisons by time for social media behavior

eTable 3. Model means and condition comparisons by time for willingness to intervene or share

eTable 4. Stimuli exposure on Invibe (passive social media exposure)

eTable 5. Platforms and topics for past week intervening

This supplemental material has been provided by the authors to give readers additional information about their work.

© 2026 Lazard AJ et al. *JAMA Network Open*.

eTable 1. Survey items for self-reported willingness to intervene and share

Construct	Item	Response
	**Message specific outcomes**	
Prompt	We will now ask you questions about three of the posts from *Invibe* on the following pages.	
Forced exposure	**[Page break.]**<br>Below is one of the posts from *Invibe*. Review this post and answer the questions below.<br><br>We will also ask you about this post on the next page of the survey.<br><br>**[Stimuli]**	
	**[Randomize the order of willingness to flag, dislike, and like.]**	
Willingness to flag	How willing are you to **flag** this post?	1 = Not at all willing<br>2 = Slightly willing<br>3 = Somewhat willing<br>4 = Moderately willing<br>5 = Extremely willing
Willingness to dislike	How willing are you to **dislike (thumbs down)** this post?	
Willingness to like	How willing are you to **like (heart)** this post?	
Willingness to comment	How willing are you to **comment** on this post?	
Comment valence	What type of comment would you write on this post?	1 = Comment to agree<br>2 = Comment to disagree<br>3 = Other, please specify: **[Text entry]**<br>4 = I would not comment
Sharing intentions	How likely are you to share or tell someone about this post?	1 = Not at all likely<br>2 = Slightly likely<br>3 = Somewhat likely<br>4 = Moderately likely<br>5 = Extremely likely
Sharing channel	**[Time 1 and 5 only]**<br>How would you share about this post? Select all that apply.	**[Multiple responses, randomize responses 1-4]**<br>1 = Private message on social media<br>2 = Text message<br>3 = In person<br>4 = Repost on your social media<br>5 = Other, please specify: **[Text entry]**<br>6 = None of these ways **[Answer exclusive]**
Sharing recipient	**[Time 1 and 5 only]**<br>Who would you share about this post with? Select all that apply.	**[Multiple responses]**<br>1 = Someone with cancer<br>2 = Someone who is a cancer caregiver<br>3 = Health-care provider<br>4 = Other, please specify: **[Text entry]**<br>5= No one **[Answer exclusive]**
Intervening behavior	**[Time 4 only]**<br>In the last 7 days, did you take action against false and potentially harmful cancer posts from social media? Select all that apply.	**[Multiple responses. Randomize responses 1-6.]**<br>1 = Yes, I muted an account(s) to stop seeing someone's posts<br>2 = Yes, I flagged a post(s)<br>3 = Yes, I disliked a post(s)

© 2026 Lazard AJ et al. *JAMA Network Open*.

		4 = Yes, I commented to correct a post(s)<br>5 = Yes, I chatted with others to disagree with post information<br>6 = Yes, I encouraged someone to flag or report a post<br>7 = Other, please tell us more: **[Text entry]**<br>8 = No, I did not do anything about false and potentially harmful cancer posts **[Answer exclusive]**
Mute – platform	[**Time 4 only. Page break. Display logic: Only if muted.]**<br>On which platform(s) did you **mute** an account to stop seeing someone's posts? Select all that apply.	**[Multiple responses. Randomize 1-7.]**<br>1 = Facebook<br>2 = Instagram<br>3 = Snapchat<br>4 = Twitter/X<br>5 = YouTube<br>6 = TikTok<br>7 = Reddit<br>8 = Other, please specify: **[Text entry]**
Mute – topic	[**Time 4 only. Display logic: Only if muted.]**<br>What was the account that you **muted** posting about? Select all that apply.	**[Multiple responses. Randomize 1-6.]**<br>1 = Alternative cancer cures (not clinically recommended)<br>2 = To adopt a new mindset<br>3 = Diet and nutrition advice<br>4 = Physical activity advice<br>5 = Advice to stop clinical care<br>6 = To seek treatment outside the US<br>7 = Other, please specify: **[Text entry]**
Flag – platform	[**Time 4 only. Page break. Display logic: Only if flagged.]**<br>On which platform(s) did you **flag** to report a post? Select all that apply.	**[Multiple responses. Randomize 1-7.]**<br>1 = Facebook<br>2 = Instagram<br>3 = Snapchat<br>4 = Twitter/X<br>5 = YouTube<br>6 = TikTok<br>7 = Reddit<br>8 = Other, please specify: **[Text entry]**
Flag – topic	**[Time 4 only. Display logic: Only if flagged.]**<br>What was the post you **flagged** about? Select all that apply.	**[Multiple responses. Randomize 1-6.]**<br>1 = Alternative cancer cures (not clinically recommended)<br>2 = To adopt a new mindset<br>3 = Diet and nutrition advice<br>4 = Physical activity advice<br>5 = Advice to stop clinical care<br>6 = To seek treatment outside the US<br>7 = Other, please specify: **[Text entry]**
Dislike – platform	[**Time 4 only. Page break. Display logic: Only if disliked.]**<br>On which platform(s) did you **dislike** a post? Select all that apply.	**[Multiple responses. Randomize 1-2.]**<br>1 = YouTube<br>2 = Reddit<br>3 = Other, please specify: **[Text entry]**

© 2026 Lazard AJ et al. *JAMA Network Open*.

Dislike – topic [DislikeTopic]	**[Time 4 only. Display logic: Only if disliked.]**<br>What was the post you **disliked** about? Select all that apply.	**[Multiple responses. Randomize 1-6.]**<br>1 = Alternative cancer cures (not clinically recommended)<br>2 = To adopt a new mindset<br>3 = Diet and nutrition advice<br>4 = Physical activity advice<br>5 = Advice to stop clinical care<br>6 = To seek treatment outside the US<br>7 = Other, please specify: **[Text entry]**
Refute – platform	[**Time 4 only. Page break. Display logic: Only if commented.]**<br>On which platform(s) did you **comment** to correct a post? Select all that apply.	**[Multiple responses. Randomize 1-6.]**<br>1 = Facebook<br>2 = Instagram<br>3 = Twitter/X<br>4 = YouTube<br>5 = TikTok<br>6 = Reddit<br>7 = Other, please specify: **[Text entry]**
Refute – topic	**[Time 4 only. Display logic: Onlly if commented.]**<br>What was the post you **commented** to correct about? Select all that apply.	**[Multiple responses. Randomize 1-6.]**<br>1 = Alternative cancer cures (not clinically recommended)<br>2 = To adopt a new mindset<br>3 = Diet and nutrition advice<br>4 = Physical activity advice<br>5 = Advice to stop clinical care<br>6 = To seek treatment outside the US<br>7 = Other, please specify: **[Text entry]**
Chat – who	[**Time 4 only. Page break. Display logic: Only if chatted.]**<br>Who did you **chat** with to disagree about post information? Select all that apply.	**[Multiple responses]**<br>1 = Someone with cancer<br>2 = Someone who is a cancer caregiver<br>3 = Health-care provider<br>4 = Other, please specify: **[Text entry]**<br>5 = No one **[Answer exclusive]**
Chat – topic	**[Time 4 only. Display logic: Only if chatted.]**<br>What was the post you **chatted** with others to disagree about? Select all that apply.	**[Multiple responses. Randomize 1-6.]**<br>1 = Alternative cancer cures (not clinically recommended)<br>2 = To adopt a new mindset<br>3 = Diet and nutrition advice<br>4 = Physical activity advice<br>5 = Advice to stop clinical care<br>6 = To seek treatment outside the US<br>7 = Other, please specify: **[Text entry]**
Encourage flag – who	[**Time 4 only. Page break. Display logic: Only if encouraged.]**<br>Who did you **encourage** to flag or report a post on social media? Select all that apply.	**[Multiple responses]**<br>1 = Someone with cancer<br>2 = Someone who is a cancer caregiver<br>3 = Health-care provider<br>4 =Other, please specify: **[Text entry]**<br>5 = No one **[Answer exclusive]**
Encourage flag – topic	**[Time 4 only. Display logic: Only if encouraged.]**	**[Multiple responses. Randomize 1-6.]**

© 2026 Lazard AJ et al. *JAMA Network Open*.

	What types of post did you **encourage** someone to flag or report? Select all that apply.	1 = Alternative cancer cures (not clinically recommended)<br>2 = To adopt a new mindset<br>3 = Diet and nutrition advice<br>4 = Physical activity advice<br>5 = Advice to stop clinical care<br>6 = To seek treatment outside the US<br>7 = Other, please specify: **[Text entry]**

© 2026 Lazard AJ et al. *JAMA Network Open*.

eTable 2. Model means and condition comparisons by time for social media behavior

	A Social cue prompt		*B Standard prompt*		*C Control, no prompt*		*A vs. C*	*B vs. C*	*A vs. B*
	M	*SE*	*M*	*SE*	*M*	*SE*	*p*	*p*	*p*
**Flagging behavior**									
Time 1	0.34	0.25	0.27	0.24	0.07	0.41	**<.001**	**.001**	.35
Time 2	0.43	0.24	0.25	0.27	0.14	0.33	**<.001**	.09	**.02**
Time 3	0.37	0.24	0.24	0.27	0.15	0.31	**.002**	.14	.10
Time 4	0.42	0.25	0.27	0.26	0.13	0.32	**<.001**	**.03**	.08
Time 5	0.35	0.25	0.27	0.26	0.14	0.32	**.003**	**.03**	.32
**Disliking behavior**									
Time 1	0.08	0.41	0.14	0.29	0.08	0.41	.97	.15	.17
Time 2	0.13	0.35	0.20	0.29	0.08	0.41	.34	**.03**	.20
Time 3	0.13	0.31	0.15	0.31	0.13	0.34	.97	.62	.64
Time 4	0.21	0.29	0.22	0.27	0.10	0.37	.07	**.05**	.91
Time 5	0.15	0.33	0.22	0.27	0.14	0.33	.79	.18	.29
**Liking behavior**									
Time 1	0.09	0.39	0.02	0.58	0.10	0.30	.84	**.008**	**.04**
Time 2	0.04	0.50	0.03	0.50	0.10	0.33	.06	**.04**	.84
Time 3	0.00	0.00	0.01	1.00	0.11	0.32	**.001**	**.002**	.32
Time 4	0.03	0.75	0.01	1.00	0.06	0.48	.37	.07	.37
Time 5	0.02	0.71	0.01	1.00	0.11	0.35	**.01**	**.004**	.50

© 2026 Lazard AJ et al. *JAMA Network Open*.

eTable 3. Model means and condition comparisons by time for willingness to intervene or share

	A Social cue prompt		*B Standard prompt*		*C Control, no prompt*		*A vs. C*	*B vs. C*	*A vs. B*
	M	*SE*	*M*	*SE*	*M*	*SE*	*p*	*p*	*p*
**Willingness to flag**									
Time 1	3.19	0.16	3.53	0.15	2.33	0.12	**<.001**	**<.001**	.12
Time 2	3.57	0.16	3.64	0.15	2.46	0.14	**<.001**	**<.001**	.76
Time 3	3.85	0.16	3.71	0.15	2.59	0.14	**<.001**	**<.001**	.50
Time 4	3.81	0.16	3.72	0.15	2.79	0.14	**<.001**	**<.001**	.70
Time 5	3.59	0.17	3.73	0.16	2.28	0.15	**<.001**	**<.001**	.55
**Willingness to dislike**									
Time 1	3.20	0.15	3.39	0.16	2.36	0.12	**<.001**	**<.001**	.38
Time 2	3.49	0.16	3.59	0.16	2.56	0.14	**<.001**	**<.001**	.67
Time 3	3.56	0.17	3.63	0.15	2.51	0.14	**<.001**	**<.001**	.78
Time 4	3.64	0.17	3.84	0.14	2.79	0.14	**<.001**	**<.001**	.37
Time 5	3.52	0.17	3.80	0.16	2.17	0.14	**<.001**	**<.001**	.23
**Willingness to like**									
Time 1	1.89	0.12	1.58	0.11	2.53	0.11	**<.001**	**<.001**	.07
Time 2	1.62	0.12	1.47	0.10	2.38	0.14	**<.001**	**<.001**	.36
Time 3	1.37	0.11	1.47	0.10	2.44	0.14	**<.001**	**<.001**	.47
Time 4	1.47	0.11	1.34	0.09	1.94	0.12	**.005**	**<.001**	.36
Time 5	1.47	0.12	1.32	0.09	2.35	0.15	**<.001**	**<.001**	.30
**Willingness to comment**									
Time 1	2.45	0.15	2.42	0.14	2.67	0.13	.27	.20	.90
Time 2	2.25	0.15	2.46	0.14	2.64	0.14	.06	.36	.32
Time 3	2.08	0.15	2.32	0.14	2.44	0.13	.07	.53	.25
Time 4	2.28	0.15	2.37	0.16	2.46	0.15	.40	.68	.69
Time 5	2.13	0.14	2.03	0.15	2.44	0.14	.13	**.05**	.61
**Willingness to share**									
Time 1	2.00	0.12	1.68	0.11	2.54	0.11	**.001**	**<.001**	**.04**
Time 2	1.79	0.12	1.71	0.11	2.35	0.14	**.004**	**.001**	.64
Time 3	1.56	0.10	1.49	0.09	2.34	0.14	**<.001**	**<.001**	.60
Time 4	1.72	0.11	1.51	0.10	2.11	0.13	**.02**	**<.001**	.17
Time 5	1.66	0.12	1.46	0.10	2.30	0.15	**.001**	**<.001**	.20

© 2026 Lazard AJ et al. *JAMA Network Open*.

eTable 4. Stimuli exposure on Invibe (passive social media exposure)

	Social cue prompt n=88	*Standard prompt n=92*	*Control, no prompt n=114*
	n (%) with exposure	*n (%) with exposure*	*n (%) with exposure*
**Time 1**			
Misinformation post 1	64 (73%)	79 (86%)	80 (70%)
Misinformation post 2	52 (59%)	63 (68%)	65 (57%)
Cancer support post	56 (64%)	73 (79%)	76 (67%)
**Time 2**			
Misinformation post 1	65 (74%)	73 (79%)	77 (68%)
Misinformation post 2	46 (52%)	54 (59%)	50 (44%)
Cancer support post	59 (67%)	71 (77%)	68 (60%)
**Time 3**			
Misinformation post 1	69 (78%)	77 (84%)	77 (68%)
Misinformation post 2	57 (65%)	68 (74%)	59 (52%)
Cancer support post	65 (74%)	75 (82%)	69 (61%)
**Time 4**			
Misinformation post 1	64 (73%)	76 (83%)	79 (69%)
Misinformation post 2	49 (56%)	56 (61%)	48 (42%)
Cancer support post	63 (72%)	71 (77%)	70 (61%)
**Time 5**			
Misinformation post 1	69 (78%)	75 (82%)	80 (70%)
Misinformation post 2	57 (65%)	68 (74%)	60 (53%)
Cancer support post	63 (72%)	71 (77%)	70 (61%)

© 2026 Lazard AJ et al. *JAMA Network Open*.

eTable 5. Platforms and topics for past week intervening

	Disliked posts *n*=89	Flagged posts *n*=79	Muted accounts *n*=58	Commented to correct posts *n*=41	Chatted with others to disagree *n*=31	Encouraged someone to flag posts *n*=30
	n, %	*n*, %	*n*, %	*n*, %	*n*, %	*n*, %
**Platforms**						
Facebook	–	37, 47%	30, 52%	15, 37%	–	–
Instagram	–	35, 44%	30, 52%	24, 59%	–	–
Twitter/X	–	40, 51%	20, 35%	17, 42%	–	–
YouTube	37, 42%	11, 14%	7, 12%	6, 15%	–	–
TikTok	–	21, 27%	20, 35%	16, 39%	–	–
Reddit	38, 43%	12, 15%	13, 22%	6, 15%	–	–
Snapchat	–	4, 5%	3, 5%	–	–	–
Other	24, 27%	3, 4%	0	0	–	–
**Recipient**						
Someone with cancer	–	–	–	–	17, 55%	12, 40%
Someone who is a cancer caregiver	–	–	–	–	16, 52%	19, 63%
Health-care provider	–	–	–	–	12, 39%	9, 30%
Other	–	–	–	–	3, 10%	3, 10%
**Topics**						
Alternative cancer cures (not clinically recommended)	53, 60%	55, 70%	39, 67%	19, 46%	18, 58%	16, 53%
Advice to stop clinical care	54, 61%	44, 56%	32, 55%	19, 46%	12, 39%	22, 73%
Diet and nutrition advice	19, 21%	25, 32%	17, 29%	20, 49%	12, 39%	9, 30%
Physical activity advice	7, 8%	6, 8%	7, 12%	9, 22%	8, 26%	4, 13%
To adopt a new mindset	9, 10%	7, 9%	11, 19%	9, 22%	4, 13%	5, 17%
To seek treatment outside the US	9, 10%	4, 5%	4, 7%	7, 17%	2, 7%	3, 10%
Other	1, 1%	1, 1%	1, 2%	0	0	0

Note: platforms and topics were select all that apply so percentages may exceed 100. Platforms were only included in the response options if those features were available at the time of the study launch. All options not shown are indicated by "–" in the table.

© 2026 Lazard AJ et al. *JAMA Network Open*.
