# Supplementary material for: User Interface Designs to Address Cancer Misinformation: A Randomized Clinical Trial
Source: JAMA Netw Open. 2026 Oct 29;9(10):e2641122. doi: 10.1001/jamanetworkopen.2026.41122 (PMC13720694; doi:10.1001/jamanetworkopen.2026.41122)
Supplement: Supplementary file 3 [file jamanetwopen-e2641122-s003.pdf]

# Data Sharing Statement

Lazard. User Interface Designs to Address Cancer Misinformation. *JAMA Netw Open*. Published October 29, 2026. doi:10.1001/jamanetworkopen.2026.41122

**Data**
**Additional Information:** This trial was pre-registered at AsPredicted #219281 (https://aspredicted.org/te3su2.pdf) on March 24, 2025, and registered on ClinicalTrials.gov (NCT07588607) on May 15, 2026.
**Data available:** Yes
**Data types:** Deidentified participant data
**How to access data:** Deidentified participant data is available here: https://doi.org/10.17615/wgsa-e207
**When available:** beginning date: 02-24-2026

**Supporting Documents**
**Document types:** Other (please specify)
**Additional Information:** Codebook provided with data.
**How to access documents:** The codebook is available now.
**When available:** beginning date: 02-24-2026

**Additional Information**
**Who can access the data:** The data is publicly available.
**Types of analyses:** For any purpose.
**Mechanisms of data availability:** The data is publicly available.
